# Exploratory identification of coding and splicing-related SNV variants in A1A1 and A2A2 β-casein Holstein dairy cows

**DOI:** 10.1093/jas/skag233

**Published:** 2026-07-29

**Authors:** Lucía Jiménez-Montenegro, Olaia Urrutia, Ángela Cánovas

**Affiliations:** Centre for Genetic Improvement of Livestock, Department of Animal Biosciences, University of Guelph, Guelph, ON N1G 2W1, Canada; Institute on Innovation and Sustainable Development in Food Chain (IS-FOOD), Public University of Navarre (UPNA), Pamplona, 31006, Spain; Centre for Genetic Improvement of Livestock, Department of Animal Biosciences, University of Guelph, Guelph, ON N1G 2W1, Canada

**Keywords:** A2 β-casein, dairy cows, milk fat globules, functional genomics, gene splicing, single nucleotide variants

## Abstract

The A2 β-casein variant has gained considerable interest in the dairy industry due to proposed health-related benefits, leading to an increasing frequency of the A2A2 genotype in dairy herds. Although the A1/A2 substitution in the β-casein gene (*CSN2*) does not directly affect gene regulation, previous transcriptomic studies have reported differences in mRNA isoform expression between A1A1 and A2A2 cows. Therefore, the objective of this study was to identify single nucleotide variants (SNVs) in A1A1 and A2A2 β-casein groups of cows using milk fat globule (MFG) RNA-seq data and to evaluate their predicted functional consequences. RNA sequencing was performed on MFG samples obtained from 14 lactating Holstein cows (A1A1, *n* = 7; A2A2, *n* = 7). Variants were classified according to their predicted effects as amino acid changing (AAC) variants, splice site effect (SSE) variants, or variants presenting both consequences. Additionally, variant data were integrated with previously reported mRNA isoform expression results, and only variants located in genes showing expression levels ≥ 0.2 FPKM were retained for further analysis. Candidate RNA-seq-derived variants differing between A1A1 and A2A2 β-casein genotype groups were identified in genes involved in mammary gland function and lactation, including mitochondrial function, lipid metabolism, vesicle trafficking and secretion, and RNA processing. Among the prioritized genes, A1A1 cows showed a greater representation of SNVs located in genes involved in mitochondrial oxidative phosphorylation (*NDUFV2*, *NDUFAB1*, *COX7A2*, and *ATP5PF*), while additional SNVs were identified in lipid metabolism-related genes (*ACSL1*, *ATP10A*, and *MFGE8*). In contrast, A2A2 group of cows showed a greater representation of SNVs located in genes involved in lipid metabolism (*LPIN1*, *FASN*, *SPTLC2*, and *ATP11B*) and vesicle trafficking and secretion (*SEC31A*, *LRRK2*, *DBNL*, *EIPR1*, and *ABCG2*). Overall, these findings provide additional insight into the molecular differences detected between A1A1 and A2A2 groups of cows. Although the predicted functional consequences of the identified variants are currently based on in silico analyses, the novel SNVs reported here constitute a valuable resource for future studies investigating the biological consequences associated with selection for the A2A2 β-casein genotype in Holstein dairy cattle.

## Teaser

Novel RNA-seq-derived SNVs were identified in A1A1 and A2A2 β-casein genotype groups, including variants located in genes relevant to mammary gland function and lactation. The results provide a resource for future studies aimed at validating and characterizing candidate variants identified in A1A1 and A2A2 β-casein genotype groups.

## Introduction

Milk from domestic dairy mammals has long been a fundamental component of the human diet and remains a nutritionally valuable food that contributes substantially to global nutrition (Huppertz 2013; Slepicka et al. 2021; Smith et al. 2021). Global milk production and overall dairy consumption have continued to increase and are expected to rise further (OECD-FAO 2017; FAO 2025). In addition, growing concern has emerged regarding undesirable health responses to cow’s milk, including cow’s milk protein allergy and milk-related intolerances (Rangel et al. 2017; Jaiswal and Worku 2022; Azani et al. 2025; Stafie 2025). Increasing attention has been directed towards the protein fraction of milk, particularly β-casein, as a potential cause of milk protein intolerance in addition to the classical lactose intolerance (Rangel et al. 2017). This intolerance has been primarily linked to the differential digestion of β-casein variants. The two most common bovine β-casein variants, A1 and A2, differ by a single amino acid substitution at position 67, histidine in A1 and proline in A2, arising from a single nucleotide variant (SNV) in the *CSN2* gene (Farrell et al., 2004; Kaskous 2020; Sebastiani et al. 2020; Jiménez-Montenegro et al. 2022; de Vasconcelos et al. 2023). This substitution results in more frequent release of the bioactive peptide β-casomorphin-7 (BCM-7) from A1 β-casein, which has been associated with gastrointestinal discomfort such as delayed transit or inflammation, whereas A2 milk appears to be better tolerated (Barnett et al. 2014; Pal et al. 2015; Milan et al. 2020; Dantas et al. 2023; Choi et al. 2024; González-Rodríguez et al. 2025).

Despite some of the uncertainties, particularly the limited number of clinical studies in humans and limited scientific evidence regarding the health effects of A1 β-casein (Barnett et al. 2014; Jiménez-Montenegro et al. 2022; Dantas et al. 2023; Gonzales-Malca et al. 2023), a trend toward selective breeding of A2A2 β-casein cows has observed (Alfonso et al. 2019; Fernández-Rico et al. 2022).

In this context, the mammary gland transcriptome of A1A1 and A2A2 β-casein cows has been assessed to determine whether β-casein genotype is associated with differential gene and mRNA isoform expression profiles during lactation (Jiménez-Montenegro et al. 2025a, 2025c). Gene level RNA-Sequencing (RNA-Seq) analyses revealed minimal expression differences between genotypes, with only two mitochondrial genes, NADH dehydrogenase subunit 6 (*ND6*) and 16S mitochondrial rRNA (*16S Mt-rRNA*), identified as differentially expressed (DE) (Jiménez-Montenegro et al. 2025a). In contrast, mRNA isoform level analyses revealed transcriptomic divergence, with 3,454 DE mRNA isoforms between A1A1 and A2A2 β-casein group of cows, primarily affecting metabolic pathways related to energy and lipid metabolism, protein folding and secretory processes (Jiménez-Montenegro et al. 2025c).

Building on these transcriptomic differences, we hypothesized that Holstein cows carrying different β-casein genotypes (A1A1 and A2A2) may also differ in the patterns of expressed functional variants present in genes relevant to mammary gland biology. RNA-Seq data provide an opportunity to identify SNVs within expressed regions of the genome, including variants predicted to alter protein sequence through amino acid changing (AAC) effects or to affect transcript processing through splice site effects (SSE) (Cánovas et al. 2010; Suárez-Vega et al. 2017; Lam et al. 2020, 2021; Jehl et al. 2021; Cunha et al. 2025). Such variants represent a potential source of functional diversity and have been successfully used to investigate the genetic basis of economically important traits in ruminants, including milk production, fertility, health, and feed efficiency (Cánovas et al. 2010; Dias et al. 2017; Suárez-Vega et al. 2017; Lam et al. 2021; Cunha et al. 2025).

Thus, this study aimed to: (i) identify SNVs located in expressed coding regions and predicted to induce AAC and/or SSE in A1A1 and A2A2 β-casein group of Holstein cows using RNA-Seq data derived from MFG; (ii) identify SNVs predicted to induce AAC with potential impact on protein function; (iii) identify SNVs predicted to induce SSE with high and moderate impact; and (iv) integrate variant information with previously reported mRNA isoform expression profiles to explore potential relationships between genetic variation and transcriptomic differences between β-casein genotype groups.

## Material and methods

### Experimental design

No experimental procedures were performed on animals for the purposes of this study. All cows belonged to a commercial dairy farm, and milk samples were obtained during routine milking using non-invasive sampling procedures. Therefore, specific ethical approval was not required. Sample collection was carried out by farm personnel in accordance with European Union Directive 2010/63/EU and Spanish Royal Decree 53/2013 on the protection of animals used for scientific purposes.

The source of the animals and the sampling process protocol were described in detail in Jiménez-Montenegro et al. (2025a, 2025b). Briefly, a total of 14 lactating healthy Holstein cows were selected for the study based on β-casein genotype (*CSN2* gene): A1A1 (*n* = 7) vs. A2A2 (*n* = 7). All cows were in the third lactation, with 225.8 ± 73.1 (mean ± SD) days in milk and an average age of 59.0 ± 3.8 months. They had similar milk production characteristics, with average milk yield, fat percentage and protein percentage of 47.8 ± 7.7 kg, 3.83 ± 0.54% and 3.31 ± 0.22% for A1A1 cows, and 47.2 ± 5.7 kg, 3.87 ± 0.55% and 3.56 ± 0.26% for A2A2 cows, respectively. The 14 cows originated from 12 different sires distributed across both β-casein genotype groups. To evaluate the potential influence of pedigree structure on the comparison between genotypes, genealogical information available for parents, grandparents, and great-grandparents was used to estimate pairwise kinship coefficients using the kinship2 package in Rstudio (version 4.3.2). Before sampling, all equipment used was disinfected with both ethanol 70% and RNaseZap (Ambion, Austin, TX, USA). Nipple cleaning was also carried out by applying both ethanol 70% and povidone iodine twice, followed by careful drying of the entire nipple. The first portion of milk was discarded before sampling to avoid collecting foremilk and potential contamination and to ensure sample representativeness. Milk was then manually collected from individual cows by the responsible farm staff and transferred into 50 mL RNase-free tubes. Approximately 20–25 tubes were collected from each animal to ensure enough MFG fraction for RNA isolation. Full milk samples were immediately snap-frozen in liquid nitrogen and transported to the laboratory, where they were stored at −80°C until further analysis.

### RNA isolation and quality assessment

RNA isolation from MFG was performed as described by Jiménez-Montenegro et al. (2025b). RNA quality and integrity were evaluated using the A260/280 by the Nanodrop 2000 spectrophotometer (Thermo Scientific, Madrid, Spain). RNA Integrity Number (RIN) was evaluated using the TapeStation 4200 RNA Screentape (Agilent Technologies, Santa Clara, CA, USA). Also, the RNA Integrity and Quality (IQ) value was evaluated using the Qubit 4 fluorometer (Life Technologies, CA, USA). RNA quality parameters (A260/280, RIN, and IQ) for A1A1 and A2A2 β-casein group of cows are further discussed in Jiménez-Montenegro et al. (2025a, 2025b). Sequencing libraries were constructed using the Illumina TruSeq stranded mRNA kit (Illumina, San Diego, CA, USA) and sequencing was completed with an Illumina NovaSeq platform (Illumina, Inc., San Diego, CA, USA) that yielded 150 base pair (bp) paired-end sequences (2 × 150 bp) (Jiménez-Montenegro et al. 2025a, 2025b).

### Variant detection and filtering

Quality control and trimming of paired-end reads were performed as described by Cánovas et al. (2014), including removal of low-quality bases, ambiguous nucleotides, adapter sequences, and the first 10 nucleotides from the 5′ end of both reads, with trimming efficiency assessed by a second quality control step. The resulting high-quality reads derived from MFG samples were subsequently mapped to the bovine reference genome (ARS.UCD.1.3). Variant detection analyses were conducted using CLC Genomics Workbench (CLC Bio Version 20.0.4, Aarhus, Denmark) as described by Cánovas et al. (2010) and Lam et al. (2020, 2021). To increase sequencing depth and improve variant detection sensitivity, reads from individuals belonging to the same β-casein genotype group were merged prior to variant calling, as previously described by Lam et al. (2020). Variants were then identified independently for the A1A1 and A2A2 groups using the Fixed Ploidy Variant Detection tool with the following parameters: ploidy = 2, minimum variant probability = 90%, minimum coverage = 10, minimum variant count = 2, minimum variant frequency = 20%, and exclusion of positions with coverage greater than 100,000. Detected SNVs were subsequently classified as AAC, splice site effect (SSE), or both using CLC Genomics Workbench (Dias et al. 2017).

### Functional annotation analysis

The Ensembl Variant Effect Predictor (VEP) (https://www.ensembl.org/vep) was used to classify the variants according to their functional impact and consequences on transcripts (McLaren et al. 2016; Dyer et al. 2025). The SNVs were selected based on high, moderate, low, or modifier functional effect, which included: missense variant, stop gain, stop loss, intergenic, 5' or 3' untranslated region, splice-donor variant, splice-acceptor variant, splice-region variant, upstream or downstream gene variant. Only variants located in expressed transcript isoforms with FPKM ≥ 0.2, as described by Mortazavi et al. (2008), were considered for downstream analyses. Priority was given to SNVs predicted to induce AAC with SIFT scores < 0.05, as well as to SNVs predicted to cause SSE with high/moderate impact located in genes containing at least one DE mRNA isoform (Jiménez-Montenegro et al. 2025c). Additionally, the number of SNVs shared between groups was assessed through Venn diagrams generated by Venny 2.1.0.

## Results

### Detection and functional classification of SNVs in A1A1 and A2A2 β-casein group of cows

A total of 149,693 SNVs were identified across both A1A1 and A2A2 β-casein group of Holstein cows, with 72,317 SNVs detected in A1A1 group and 77,376 SNVs in A2A2 group. After classification of variant consequences and integration with transcript expression data (FPKM ≥ 0.2), 909 and 1,409 SNVs were predicted to induce AAC, and 104 and 117 SNVs were predicted to cause SSE in A1A1 and A2A2 groups, respectively. Additionally, 86 SNVs in A1A1 group and 68 SNVs in A2A2 group were predicted to exhibit both AAC and SSE effects (Table 1). At the transcript level, 1,748 and 2,409 expressed transcript isoforms (FPKM ≥ 0.2) containing SNVs predicted to induce AAC were identified in the A1A1 and A2A2 groups, respectively. Similarly, 225 and 216 expressed transcript isoforms contained SNVs predicted to cause SSE in the A1A1 and A2A2 groups, respectively.

**Table 1:** Number (*n*) of single nucleotide variants (SNVs) with predicted functional consequences in expressed genes of A1A1 and A2A2 β-casein group of cows (≥ 0.2 FPKM). Variants were classified according to their predicted functional consequences as inducing amino acid changes (AAC), splice site effects (SSE), or both effects simultaneously (AAC + SSE).

Group	AAC (*n*)	SSE (*n*)	Both (AAC + SSE) (*n*)
**A1A1**	909	104	86
**A2A2**	1,409	117	68
**Shared A1A1/A2A2^1^**	10	0	0

1 Indicate variants common in both genotypes (AAC, SSE, or AAC + SSE).

Meanwhile, this study revealed a limited number of SNVs shared between A1A1 and A2A2 groups, indicating that most of the functionally annotated variants identified were detected specifically in one genotype group under the present RNA-seq conditions. Among the ten SNVs detected in both A1A1 and A2A2 groups, seven were identified within transcripts corresponding to novel genes. Regarding the remaining shared variants, the SNV located at position 4:113263951, resulted in a missense variant with moderate predicted impact in the transcript GIMAP6-201 in both groups. Another shared SNV was found within the *ANKS1A* gene, at position 23:9056733, resulting in a missense variant with moderate impact; however, the number of affected transcripts differed between genotypes, with four transcripts in A1A1 group (ANKS1A-201, ANKS1A-202, ANKS1A-203, and ANKS1A-204) and only one transcript in A2A2 group (ANKS1A-204). Finally, one shared SNV, located at position 23:27829922, was associated with both a bovine microRNA (bta-miR-10167) and a transcript of the *BoLA* gene (BoLA-201). This variant was annotated as a missense variant with moderate impact for *BoLA* gene, while it was classified as an upstream gene variant with low predicted functional relevance for the microRNA.

**Figure 1:**
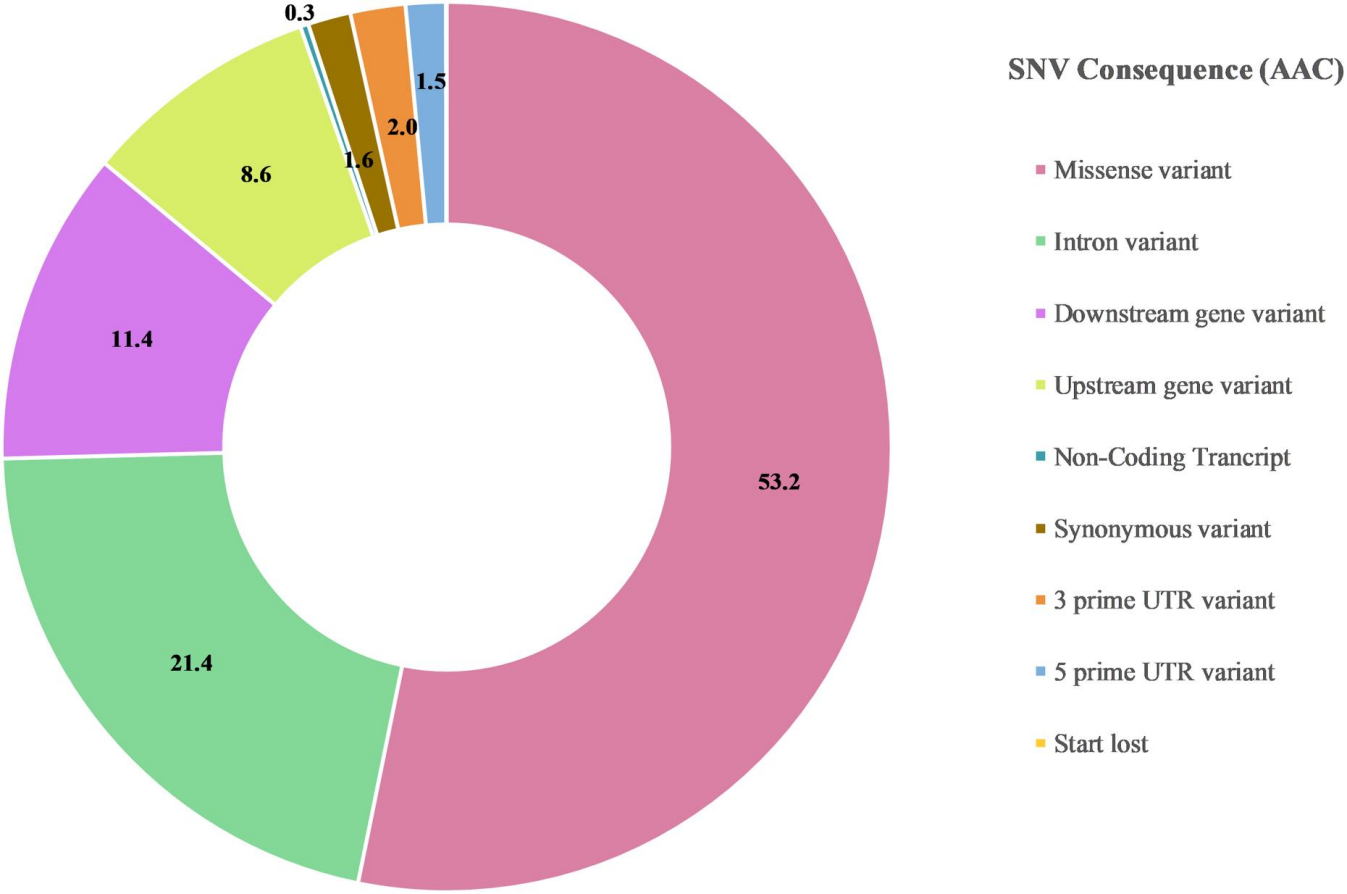
Distribution of amino acid changing (AAC) SNVs detected in expressed transcripts (FPKM ≥ 0.2) of A1A1 and A2A2 β-casein groups of cows according to predicted VEP consequence categories.

**Figure 2:**
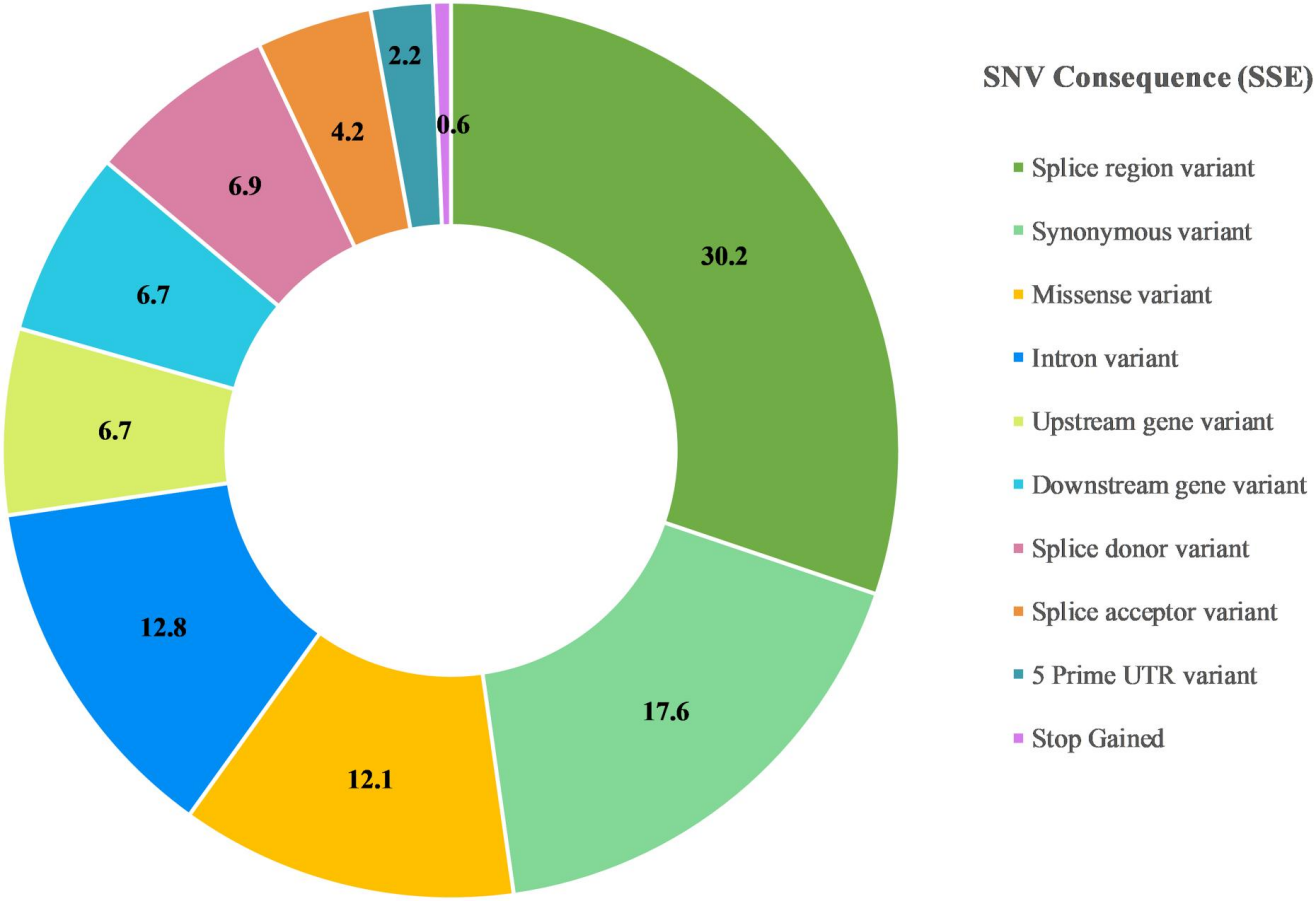
Distribution of splice site effect (SSE) SNVs detected in expressed transcripts (FPKM ≥ 0.2) of A1A1 and A2A2 β-casein groups of cows according to predicted VEP consequence categories.

The percentage of variants within each predicted consequence type is described in Figures 1 and 2. Among SNVs predicted to induce AAC (Figure 1), the predominant consequence was missense variant (53.2%), followed by intron variant (21.4%), downstream gene variant (11.4%) and upstream gene variant (8.6%). The remaining consequence types each represented less than 2% of the annotated variants. Missense variants are sequence variants that change one or more bases within the coding region, resulting in an altered amino acid sequence while preserving the overall length of the protein. According to VEP classification, missense variants are generally considered to have a moderate predicted impact, as their effects on protein structure and function may range from benign to pathogenic and depend on the properties and localization of the altered amino acid within the protein (Kamburov et al. 2015; Lek 2016; Sivley et al. 2018; Iqbal et al. 2020). Moreover, upstream and downstream gene variants refer to sequence changes located before the 5′ end and after the 3′ end of a gene, respectively. These variants are classified as having a “modifier” impact, as they occur outside protein-coding regions or within non-coding genes, and their functional consequences are often difficult to predict or remain poorly characterized. Likewise, intron variants correspond to sequence changes within intronic regions and are also generally assigned a modifier impact (Cunha et al. 2025).

Regarding SNVs predicted to cause SSE (Figure 2), the most frequent consequence was splice region variant (30.2%), followed by synonymous variant (17.6%), intron variant (12.8%), and missense variant (12.1%). Splice-region variants correspond to sequence changes occurring near splice sites, typically within 1–3 bases of the exon or 3–8 bases of the intron, and are generally classified as having a “low” predicted impact, as they are considered unlikely to substantially affect the final protein product. Although less frequent, splice-donor variants (6.9%) and splice-acceptor variants (4.2%) were also identified and are of particular relevance due to their location within the conserved splice site dinucleotides at the 5′ and 3′ ends of introns, respectively, resulting in their classification as high-impact variants. Finally, stop-gained variants represented only 0.6% of the detected SNVs but were also classified as having a high predicted impact because they introduce premature termination codons, which may result in truncated proteins. Definitions of variant consequences and impact categories were based on the software annotation framework (McLaren et al. 2016).

Although variants were detected from RNA-seq reads, their occurrence is not necessarily associated with differential transcript expression or transcript abundance in the tissue (Cánovas et al. 2010; Lam et al. 2021). Accordingly, variants were prioritized according to their predicted functional consequences and the biological relevance of the affected genes to mammary gland function and milk synthesis. Information on differentially expressed mRNA isoforms was used as complementary evidence when available, particularly for variants predicted to induce SSE.

### Predicted functional impact of SNVs inducing amino acid changes (ACC)

As shown in Table 1, a total of 909 SNVs in the A1A1 group and 1,409 SNVs in the A2A2 group were identified in expressed transcripts (FPKM ≥ 0.2). These variants were distributed across 1,748 and 2,409 expressed transcript isoforms in the A1A1 and A2A2 groups, respectively. Given the large number of variants and affected transcripts, only SNVs showing a predicted SIFT score < 0.05 were retained for further analysis. SIFT predicts the potential impact of amino acid substitutions on protein function based on sequence conservation, with scores below 0.05 indicating variants likely to affect protein function (Wang et al. 2022). Following this filtering step, 391 transcript isoforms in the A1A1 group and 353 transcript isoforms in the A2A2 group contained missense SNVs with SIFT scores < 0.05, corresponding to 234 and 214 unique genes, respectively. All SNVs identified with SIFT values < 0.05 were classified by VEP as having a moderate predicted impact, with the primary consequence being a missense variant and, in some cases, an additional splice-region variant annotation. From this set, transcripts with functional relevance to mammary gland physiology and lactation were prioritized in A1A1 and A2A2 groups (Tables S1 and S2, respectively). Additionally, SNVs located in transcripts with no detectable expression in the present dataset (< 0.2 FPKM) were reported in Tables S3 and S4. These annotations represent theoretical consequences and are provided to document the full spectrum of potential SNV effects independently of transcript expression levels.

As shown in Table S1, missense SNVs were detected in transcripts related to mitochondrial oxidative phosphorylation (OXPHOS) and the electron transport chain (ETC) in the A1A1 group. Specifically, the missense SNV 24_41416489_G/A was identified in the NDUFV2-203 transcript and the SNV 25_21261249_G/A was located in NDUFAB1-201, both encoding subunits of NADH: ubiquinone oxidoreductase (Complex I) (Tetaud et al. 2014; Dorji et al. 2020; Song et al. 2021). In addition, the missense SNV 9_14835368_T/G, located within the coding region of *COX7A2*, was identified in two transcripts (COX7A2-201 and COX7A2-202), both related to cytochrome c oxidase (Complex IV) (Deng et al. 2018). Finally, the SNV 1_10691073_T/A was detected in the ATP5PF-201 isoform, which is part of the ATP synthase complex (Complex V) (Tetaud et al. 2014; Feichtinger et al. 2018; Franco et al., 2020). None of the transcripts in which these SNVs were detected showed significant differential expression between the A1A1 and A2A2 groups under the experimental conditions evaluated in the present study. Therefore, the potential biological relevance of these variants arises from their predicted effects on protein sequence and function (SIFT < 0.05).

Beyond energy metabolism, several missense SNVs were identified in transcripts related to mitochondrial integrity and function. The missense SNV 17_70324754_A/G was identified in the *PISD* gene (PISD-205 transcript), which plays a major role in mitochondrial phospholipid synthesis. The *IMMT* gene, associated with the structure of the inner mitochondrial membrane (IMM), contained two SNVs in IMMT-203 transcript: the SNV 11_48689263_C/T and the SNV 11_48714033_G/T.

In terms of protein synthesis, the *ESF1* gene, which is involved in ribosome biogenesis, contained the missense SNV 13_7321761_C/T in ESF1-201 which in addition, was down-regulated in the A2A2 group (FDR = 0.032).

With respect to lipid metabolism, the missense SNV 27_15192468_C/T was identified in multiple transcripts of the *ACSL1* gene involved in fatty acid activation. Specifically, these transcripts were: ACSL1-202, ACSL1-203, ACSL1-204, ACSL1-205, and ACSL1-206. In addition, a missense SNV in *ATP10A* gene (21_2797002_G/C) was identified in the transcripts ATP10A-201 and ATP10A-202.

Regarding peroxisomal metabolism, two SNVs were identified in transcripts of the *PEX7* gene, which is involved in peroxisomal enzyme import. The SNV 9_74805010_C/A was located in the transcript PEX7-201, whereas the SNV 9_74833653_G/A was identified in PEX7-201 and PEX7-204 transcripts.

Additional missense SNVs were identified in genes involved in other cellular processes relevant to mammary gland function, including autophagy, RNA processing, ribosome biogenesis, apoptosis and cytoskeletal organization. The missense SNV 17_69083107_G/A was identified in the *MTMR3* gene (MTMR3-204 transcript), a negative regulator of autophagy. Moreover, the SNV 1_1979297_G/A was identified in the *SON* gene and was present in the SON-201, SON-205, and SON-206 transcripts. Notably, SON-201 was up-regulated, whereas SON-206 was down-regulated in the A2A2 group. In terms of RNA and protein synthesis, the *ESF1* gene, which is involved in ribosome biogenesis, contained the missense SNV 13_7321761_C/T in ESF1-201, which was down-regulated in the A2A2 group (FDR = 0.032). Regarding cellular stress and signaling pathways, the SNV 21_69229824_G/A was detected in SIVA1-201, a transcript associated with pro-apoptotic signaling, which was down-regulated in the A2A2 group (FDR = 0.046). In the *CARMIL1* gene, related to actin cytoskeleton dynamics, the SNV 23_32397444_T/C caused both a missense variant and a splice-region variant with moderate impact in transcripts CARMIL1-201, CARMIL1-203, and CARMIL1-204. In addition, the isoform CARMIL1-203 was up-regulated in the A2A2 group (FDR = 0.019).

As observed in the A1A1 group, missense variants were also detected in the A2A2 group within genes involved in mitochondrial integrity and function (Table S2). Within this category, two missense variants were identified in the *OPA1* gene, which plays a central role in mitochondrial morphology and cristae organization. The variant 1_73849107_G/C was detected in the OPA1-201 and OPA1-204 transcripts, whereas the variant 1_73851821_C/T was identified in OPA1-201, OPA1-204, OPA1-206, and OPA1-208. Moreover, the SNV 10_48144355_C/T was predicted to induce a missense variant in the *VPS13C* gene, which is involved in mitochondrial quality control and organelle homeostasis, specifically in the VPS13C-203 transcript. Furthermore, the SNV 17_62634248_G/T was identified in the *SIRT4* gene, involved in mitochondrial metabolic regulation, specifically in the SIRT4-201 transcript.

Similarly, missense variants in transcripts related to lipid metabolism were identified in the A2A2 group (SIFT < 0.05). Among them, the SNV 11_86097586_C/T was detected in the LPIN1-201 transcript, which is involved in triacylglycerol (TAG) synthesis. Likewise, the SNV 19_50791479_C/T was identified in the FASN-202 transcript, associated with de novo fatty acid synthesis. In addition, the missense variant 10_88784402_C/T was detected in the SPTLC2-201 transcript, involved in sphingolipid biosynthesis. Finally, the missense SNV 1_84188257_G/A was identified in the ATP11B-208 transcript, which participates in membrane phospholipid asymmetry. None of these missense variants were associated with differential expression, as indicated by FDR values ≥ 0.05.

Furthermore, several SNVs in the A2A2 group were detected in transcripts related to proteostasis and protein turnover. Specifically, the missense SNV 10_76680000_T/C was identified in the HSPA2-203 transcript, whereas the SNV 23_27525598_A/G was detected in the HSPA1L-201 and HSPA1L-204 transcripts. Both *HSPA2* and *HSPA1L* encode molecular chaperones involved in stress-responsive protein folding.

Notably, several SNVs were predicted to caused missense variants in genes linked to membrane organization and vesicle trafficking. Among them, the SNV 6_97694187_T/C was identified in the *SEC31A* gene, which is involved in ER-to-Golgi transport, and was detected in the SEC31A-201, SEC31A-203, SEC31A-205, and SEC31A-206 transcripts. Furthermore, two missense SNVs were identified within the *LRRK2* gene: 5_40643662_A/G and 5_40646293_C/T both located in transcripts LRRK2-201. In addition, the transcript DBNL-204, which participates in actin-dependent endocytosis, contained the SNV 22_380933_A/G, predicted as both a missense and splice-region variant.

Finally, regarding cellular signaling, two SNVs were identified in the RICTOR-205 transcript of the *RICTOR* gene, which is involved in mTORC2 signaling and metabolic regulation. The variant 20_35421065_C/G was annotated as both a missense and a splice-region variant, whereas 20_35421069_T/A was annotated only as a missense variant.

### Integration of SNVs predicted to induce splice site effects (SSE) with differentially expressed (DE) mRNA isoforms

As shown in Table 1, a total of 104 and 117 SNVs were predicted to cause SSE in the A1A1 and A2A2 β-casein groups, respectively. These variants were identified in 225 and 216 expressed transcript isoforms (FPKM ≥ 0.2) in the A1A1 and A2A2 groups, respectively. Given the large number of affected transcripts, only SNVs annotated by VEP as having High or Moderate predicted impact were retained for further analysis. Following this filtering step, 73 transcript isoforms in the A1A1 group and 78 transcript isoforms in the A2A2 group contained SSE variants with High or Moderate predicted impact, corresponding to 37 and 46 unique genes, respectively. Among the SSE variants with high or moderate predicted impact, those located in genes containing at least one differentially expressed mRNA isoform are presented in Tables S5 and S6 for the A1A1 and A2A2 β-casein groups, respectively. Additional VEP annotations corresponding to non-expressed transcripts containing the SNVs detected are provided in Tables S7 and S8.

In the A1A1 group, a splice-donor SNV with predicted high impact was identified in the *AIFM2* gene, specifically in the transcript AIFM2-201 (28_26347419_A/G). This variant is located at the 5′ end of an intron and may disrupt normal exon-intron recognition, potentially leading to alternative splicing outcomes such as exon skipping or intron retention. In addition, several novel mRNA isoforms of *AIFM2* previously identified (AIFM2_3, AIFM2_4, AIFM2_8, and AIFM2_9) overlapped the genomic region containing this SNV, although they are not currently annotated in Ensembl. Among these isoforms, only AIFM2_4 showed differential expression, being up-regulated in A2A2 group (FDR = 0.034).

The *CARMIL1* gene contained a SNV previously identified as a missense variant (SIFT< 0.05), also showed the predicted consequence of splice-region variant. Two novel mRNA isoforms of this gene (CARMIL1_5 and CARMIL1_6), overlapped the genomic region in which this SNV was located. Moreover, two mRNA isoforms (CARMIL1_2, FDR = 0.034, and CARMIL1-203, FDR = 0.019) showed differential expression between groups, being up-regulated in A2A2 group.

The *MFGE8* gene, which encodes a structural component of the MFG membrane (MFGM), showed a splice-donor SNV with a high predicted impact on the coding sequence. This variant was located in the expressed transcripts (MFGE8-201 and MFGE8-204). In addition, two novel mRNA isoforms (MFGE8_2 and MFGE8_3) include the genomic region where this SNV was located; however, as these isoforms were not annotated in the reference assembly, the variant was not explicitly assigned to them. Among these isoforms, MFGE8_3 was up-regulated in A2A2 group (FDR = 0.003).

The *YIF1A* gene, involved in Golgi vesicular trafficking, also presented a splice-donor SNV with high predicted impact located in the expressed transcript YIF1A-201. This variant could also be contained within novel transcripts detected YIF1A_2, YIF1A_3, and YIF1A_4, being this latter down-regulated in A2A2.

In the A2A2 group, the *ABCG2* gene, an ATP-dependent efflux transporter that mediates the secretion of small molecules from mammary epithelial cells (MEC) into milk, was found to contain a splice-acceptor SNV (6_36566686_G/A) in the annotated transcripts ABCG2-202 and ABCG2-204. The mRNA isoform-level RNA-Seq analysis identified four novel ABCG2 mRNA isoforms not previously annotated in Ensembl, among which ABCG2_3 and ABCG2_4 overlap the genomic region containing this SNV. In addition, the novel isoform ABCG2_1 was up-regulated in A2A2 (FDR = 0.023).

The splice-donor SNV 8_111160250_G/T, located within the coding region of the *EIPR1* gene, was detected in the expressed transcripts EIPR1-203 with a predicted high impact. Integration with mRNA isoform expression data identified the co-occurrence of this splice site-related variant and the down-regulation of the EIPR1-203.

Another splice-donor SNV (1_38360664_G/C) was detected in *NSUN3* gene, which is related to mitochondrial function, specifically in the annotated transcript NSUN3-201. In addition, several novel mRNA isoforms identified in the isoform-level RNA-Seq analysis (NSUN3_3 to NSUN3_6) span the genomic region containing this SNV. Among these, NSUN3_6 was up-regulated in A2A2 group (FDR = 0.035).

Finally, the *EHMT2* gene, which plays a crucial role in epigenetic repression, contained a splice-donor SNV predicted to alter the 5′ end of an intron and classified as having a high predicted impact. This variant was detected in transcripts EHMT2-201, EHMT2-202, and EHMT2-203. In addition, two novel mRNA isoforms (EHMT2_1 and EHMT2_2) encompassed the genomic region containing this SNV. Notably, EHMT2-202 was down-regulated in the A2A2 group (FDR = 0.038).

**Figure 3:**
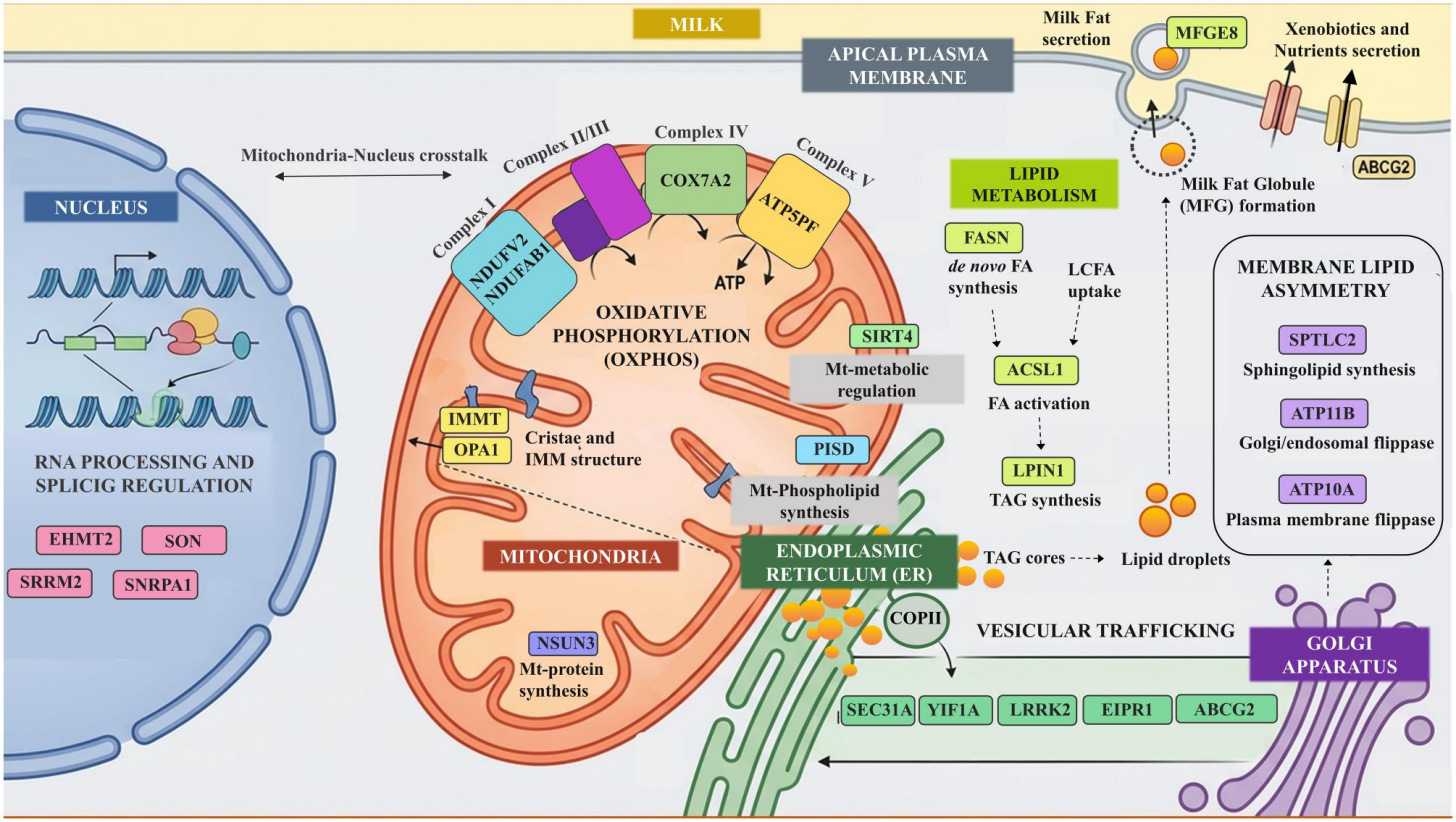
Overview of cellular processes associated with SNVs identified in A1A1 and A2A2 β-casein groups. *ABCG2*, ATP-binding cassette subfamily G member 2; *ACSL1*, Acyl-CoA synthetase long-chain family member 1*; ATP10A*, ATPase phospholipid transporting 10A; *ATP11B*, ATPase phospholipid transporting 11B; *ATP5PF*, ATP synthase peripheral stalk subunit F; *COX7A2*, Cytochrome c oxidase subunit 7A2; COPII, Coat protein complex II; *EHMT2*, Euchromatic histone lysine methyltransferase 2; ER, Endoplasmic reticulum; FA, Fatty acids; *FASN*, Fatty acid synthase; *IMMT*, Inner mitochondrial membrane protein (mitofilin); LCFA, Long-chain fatty acids; *LPIN1*, Lipin 1; *LRRK2*, Leucine-rich repeat kinase 2; MFG, Milk fat globule; *MFGE8*, Milk fat globule EGF factor 8; *NSUN3*, NOP2/Sun RNA methyltransferase family member 3; *NDUFAF2*, NADH dehydrogenase (ubiquinone) complex I assembly factor 2; *NDUFAB1*, NADH dehydrogenase (ubiquinone) subunit AB1; *OPA1*, Optic atrophy 1; OXPHOS, Oxidative phosphorylation; *PISD*, Phosphatidylserine decarboxylase; *SEC31A*, SEC31 homolog A, COPII coat complex component; *SIRT4*, Sirtuin 4; *SON*, SON DNA and RNA binding protein; SPTLC2, Serine palmitoyltransferase long-chain base subunit 2; TAG, Triacylglycerol; *YIF1A*, Yip1 interacting factor homolog.

In summary, the co-occurrence of the detected SNV and differential isoform expression within the same genomic region highlights a potentially relevant observation that warrants further investigation. To facilitate the biological interpretation, Figure 3 presents an integrative overview of the SNVs detected in the A1A1 and A2A2 β-casein group of cows, highlighting the main cellular processes and biological pathways represented by genes in which AAC and SSE SNVs were identified.

## Discussion

The integrative overview presented in Figure 3 revealed that genes containing AAC and SSE SNVs clustered into several interconnected pathways related to mitochondrial function, lipid metabolism, membrane organization, vesicular trafficking, and RNA processing in mammary epithelial cells.

Mitochondrial function represented one of the main biological modules identified in the integrative overview. Lactation constitutes a major metabolic challenge for dairy cows, requiring large amounts of energy to support milk protein, lipid, and lactose synthesis, together with substantial endocrine and metabolic adaptations (Mowry et al. 2017; Favorit et al. 2021; Álvarez-Delgado 2022). Consequently, mitochondrial oxidative metabolism plays a central role in maintaining mammary gland function during lactation (Bionaz et al. 2012; Cui et al. 2014; Favorit et al. 2021). In this context, in A1A1 group of cows, several missense SNVs predicted to affect protein function (SIFT < 0.05) were identified within genes encoding components of the oxidative phosphorylation (OXPHOS) machinery. Specifically, the SNVs 24_41416489_G/A and 25_21261249_G/A were detected in the NDUFV2-203 and NDUFAB1-201 transcripts, respectively, both encoding subunits of mitochondrial NADH oxidoreductase (Complex I) (Tetaud et al. 2014; Dorji et al. 2020; Song et al. 2021). Likewise, the SNV 9_14835368_T/G was identified in transcripts of the *COX7A2* gene, which encodes a subunit of cytochrome c oxidase (Complex IV), the enzyme responsible for the terminal step of electron transfer to molecular oxygen (Deng et al. 2018). In addition, the SNV 1_10691073_T/A was detected in the ATP5PF-201 transcript, encoding a subunit of the F_0_ domain of ATP synthase (Complex V), a key component of mitochondrial ATP production (Feichtinger et al. 2018; Franco et al. 2020; Zeng et al. 2025).

The OXPHOS system critically depends on the maintenance of the proton gradient across the inner mitochondrial membrane (IMM), which in turn requires the preservation of mitochondrial structure, membrane integrity, and mitochondrial gene expression. In this context, in A2A2 group of cows, several SNVs were located in genes involved in mitochondrial organization and regulation, including *OPA1*, *VPS13C*, *SIRT4*, and *NSUN3.* Two missense variants (1_73849107_G/C and 1_73851821_C/T) were identified in *OPA1* gene, a key regulator of mitochondrial cristae organization (Anand et al. 2014; Kondadi et al. 2020; Lin et al. 2023), while missense variants were also detected in *VPS13C* (10_48144355_C/T) and *SIRT4* (17_62634248_G/T), genes involved in mitochondrial quality control and metabolic regulation, respectively (Laurent et al. 2013; Lesage et al. 2018; Li 2018; Hancock-Cerutti et al. 2022). In addition, a splice-donor SNV (1_38360664_G/C) was detected in the NSUN3-201 transcript. *NSUN3* gene encodes a mitochondrial RNA methyltransferase required for efficient mitochondrial protein synthesis (Haag et al. 2016; Murakami et al. 2023).

Lipid metabolism represented another major functional module identified in the present study. Notably, a greater number of potentially functional SNVs were detected in A2A2 cows within genes involved in fatty acid synthesis, triacylglycerol formation, and membrane lipid metabolism. In contrast, A1A1 cows showed variants in a smaller number of genes, primarily related to fatty acid activation and MFGM structure. A notable finding in the A1A1 group was the detection of a missense SNV within multiple ACSL1 transcripts (ACSL1-202, ACSL1-203, ACSL1-204, ACSL1-205, and ACSL1-206). *ACSL1* catalyzes the activation of long-chain fatty acids to acyl-CoA derivatives, a key step linking fatty acid uptake with downstream lipid metabolic pathways (Bionaz and Loor 2008; Tian et al. 2022). Once activated, fatty acids can be channeled towards TAG synthesis, the major storage form of lipids in milk. In this pathway, LPIN1 catalyzes the conversion of phosphatidic acid to diacylglycerol, a critical intermediate in TAG formation (Du et al. 2021; Zhou et al. 2022; Guo et al. 2024). Interestingly, a missense SNV was detected in LPIN1-201 in the A2A2 group, while the novel isoform LPIN1_1 was previously found to be up-regulated in A2A2 cows (FC = 426.95; FDR = 0.011) (Jiménez-Montenegro et al. 2025c). In addition to TAG synthesis, de novo fatty acid synthesis was also represented through the detection of a missense SNV in FASN-202 in the A2A2 group. *FASN* encodes fatty acid synthase, the enzyme responsible for the synthesis of palmitate from acetyl-CoA and malonyl-CoA, providing an important source of fatty acids for milk fat production (Gao et al. 2013; Tian et al. 2022; Zhang et al. 2025).The TAG cores generated through these pathways are assembled into lipid droplets and subsequently enveloped by the apical plasma membrane of mammary epithelial cells to form MFG (Lemay et al. 2013; Lopez 2020; Jiménez-Montenegro et al. 2025b). This process depends on the proper formation and composition of the MFGM. In this context, *MFGE8*, which encodes a structural component of the MFGM (Nie et al. 2024), contained a high-impact splice-donor SNV in the A1A1 group affecting transcripts MFGE8-201 and MFGE8-204. Interestingly, the novel isoform MFGE8_3 was previously found to be up-regulated in A2A2 cows (FDR = 0.003) (Jiménez-Montenegro et al. 2025c).

Beyond lipid synthesis, efficient milk fat secretion requires the coordinated assembly, packaging and transport of cellular components through specialized secretory pathways. In agreement with this, vesicle trafficking and secretion represented another functional module strongly represented under the present RNA-seq conditions (Figure 3), particularly in A2A2 cows. Interestingly, previous studies have suggested differences in the organization and secretion of milk components between β-casein variants. The A2 β-casein variant has been associated with smaller casein micelles (Raynes et al. 2015; Nguyen et al. 2018; Daniloski et al. 2022). Comparative proteomic studies have also reported differences in the protein cargo detected across milk fractions between A1A1 and A2A2 milks, suggesting that the packaging of milk components may differ between β-casein variants (Wang et al. 2021). Furthermore, previous transcriptomic analyses suggested a coordinated adjustment of the mammary epithelial cell secretory machinery in A2A2 cows, including ER stress responses and increased vesicular transport capacity (Jiménez-Montenegro et al. 2025c). Consistent with these observations, several potentially functional variants were identified in A2A2 cows within genes involved in ER-Golgi transport, vesicle trafficking, and secretory processes. Among them, missense variants (SIFT < 0.05) were detected in *SEC31A* and *LRRK2*, two genes involved in intracellular vesicle transport. *SEC31A* encodes a structural component of COPII vesicles required for ER-Golgi trafficking, whereas *LRRK2* has been implicated in the regulation of vesicular transport pathways (Eguchi et al. 2018; Bonet-Ponce et al. 2020; Nie and Ma 2025). Additionally, high-impact splice site variants were identified in *ABCG2* and *EIPR1*. Specifically, a splice-acceptor variant was detected in ABCG2, which encodes an ATP-dependent transporter involved in the secretion of multiple compounds into the mammary lumen (Wang et al. 2012; Otero et al. 2016; García-Lino et al. 2019), whereas a splice-donor variant was identified in *EIPR1*, a gene involved in endosomal cargo sorting and secretory vesicle trafficking. Notably, both variants affect conserved splice site dinucleotides required for correct mRNA processing and are therefore predicted to have a substantial impact on transcript structure and function (McLaren et al. 2016).

RNA processing and splicing regulation represented another biological module identified in the present study. In A1A1 genotype cows, missense variants were detected in *SON and SRRM2*. *SON* is a serine/arginine-rich protein involved in nuclear speckle organization and pre-mRNA splicing, and experimental evidence indicates that *SON* depletion can alter alternative splicing patterns without necessarily affecting overall transcript abundance (Sharma et al. 2011). Similarly, *SRRM2* functions as a core component of nuclear speckles and contributes to spliceosome organization. Interestingly, *SON* was also represented in the previous mRNA isoform analysis, where the SON-206 isoform was down-regulated in A2A2 cows (Jiménez-Montenegro et al. 2025c). In contrast, A2A2 group of cows showed a high-impact splice-donor variant in *EHMT2* together with missense variants in *SNRPA1. SNRPA1* forms part of the spliceosome machinery required for pre-mRNA processing, whereas *EHMT2* has been linked to chromatin-mediated regulation of alternative splicing, as histone modifications can influence splice-site recognition and exon inclusion (Salton et al. 2014; Fiszbein et al., 2016). Notably, the EHMT2-202 isoform was also previously identified as down-regulated in A2A2 cows (Jiménez-Montenegro et al. 2025c). Notably, in addition to variants predicted to affect protein sequence or splice sites directly, candidate variants were also detected in genes involved in the regulation of RNA processing and alternative splicing, further emphasizing the representation of splicing-related mechanisms among the identified candidate variants.

Overall, these findings indicate that candidate RNA-seq-derived variants differing between A1A1 and A2A2 β-casein genotype groups are located in genes involved in key biological processes required for lactation, including mitochondrial function, lipid metabolism, mammary secretion, vesicular trafficking, and RNA processing. However, these results should be interpreted with caution, as the predicted functional consequences of the identified variants are currently based on in silico analyses. Nevertheless, they provide a valuable resource for future analysis, as the novel SNVs identified in the present study could be incorporated into DNA-based genotyping platforms or targeted sequencing approaches and evaluated in larger cattle populations to validate their distribution across β-casein genotype groups. Such studies may help to provide further insight into the genetic variation associated with selection for the A2A2 β-casein genotype in Holstein dairy cattle.

## Conclusion

This study analyzed SNVs identified from MFG RNA-seq data obtained from Holstein cows belonging to A1A1 and A2A2 β-casein genotype groups.

Under the present RNA-seq conditions, candidate RNA-seq-derived variants differing between A1A1 and A2A2 β-casein genotype groups were identified in genes involved in mitochondrial function, lipid metabolism, vesicle trafficking and secretion, and RNA processing. Among the prioritized variants, A1A1 cows showed SNVs in genes related to lipid metabolism (*ACSL1*, *ATP10A*, and *MFGE8*) and a greater representation of SNVs located in genes involved in mitochondrial oxidative phosphorylation (*NDUFV2*, *NDUFAB1*, *COX7A2*, and *ATP5PF*). In contrast, A2A2 cows showed a greater representation of SNVs located in genes involved in lipid metabolism (*LPIN1*, *FASN*, *SPTLC2*, and *ATP11B*) and vesicle trafficking and secretion (*SEC31A*, *LRRK2*, *DBNL*, *EIPR1*, and *ABCG2*). Overall, these findings provide additional insight into the molecular differences detected between A1A1 and A2A2 cows. Although the predicted functional consequences of the identified variants are currently based on in silico analyses, the novel SNVs reported here constitute a valuable resource for future studies investigating the biological consequences associated with selection for the A2A2 β-casein genotype in Holstein dairy cattle. Overall, these findings are consistent with previously reported transcriptomic differences and highlight biological pathways potentially relevant in the context of selective breeding for the A2A2 β-casein genotype in Holstein dairy cows. However, the functional consequences of these variants are currently based on in silico predictions and therefore require further experimental validation.

## Supplementary Material

skag233_Supplementary_Data

## Data Availability

The datasets generated and/or analyzed during the current study are available in the Gene Expression Omnibus (GEO) repository under the accession number: GSE273229. These can be accessed through the NCBI GEO database at https://www.ncbi.nlm.nih.gov/geo/info/seq.html.

## Abbreviations

AAC: amino acid change
ABCG2: ATP binding cassette subfamily G member 2
ACSL1: acyl-CoA synthetase long-chain family member 1
AIFM2: apoptosis inducing factor mitochondria associated 2
ATP5PF: ATP synthase peripheral stalk subunit F
ATP10A: ATPase phospholipid transporting 10A
ATP11B: ATPase phospholipid transporting 11B
CARMIL1: capping protein regulator and myosin 1 linker 1
COX7A2: cytochrome c oxidase subunit 7A2
DBNL: drebrin like protein
DE: differentially expressed
EHMT2: euchromatic histone lysine methyltransferase 2
ESF1: ESF1 ribosome biogenesis factor
ETC: electron transport chain
FASN: fatty acid synthase
FDR: false discovery rate
FPKM: fragments per kilobase per million mapped reads
HSPA1L: heat shock protein family A (Hsp70) member 1-like
HSPA2: heat shock protein family A (Hsp70) member 2
IMM: inner mitochondrial membrane
IMMT: inner mitochondrial membrane protein (mitofilin)
LPIN1: lipin 1
LRRK2: leucine rich repeat kinase 2
MEC: mammary epithelial cell
MFGE8: milk fat globule EGF factor 8
MFG: milk fat globule
MFGM: milk fat globule membrane
MTMR3: myotubularin related protein 3
NDUFAB1: NADH dehydrogenase [ubiquinone] 1 alpha subcomplex assembly factor 1
NDUFV2: NADH dehydrogenase [ubiquinone] flavoprotein 2
NMD3: nuclear export adaptor protein NMD3
NSUN3: NOP2/Sun RNA methyltransferase family member 3
OPA1: optic atrophy 1
OXPHOS: oxidative phosphorylation
PEX7: peroxisomal biogenesis factor 7
PISD: phosphatidylserine decarboxylase
RICTOR: RPTOR independent companion of MTOR complex 2
SEC31A: SEC31 homolog A, COPII coat complex component
SIFT: sorting intolerant from tolerant
SIRT4: sirtuin 4
SIVA1: SIVA apoptosis inducing factor 1
SNV: single nucleotide variant
SON: SON DNA and RNA binding protein
SPTLC2: serine palmitoyltransferase long chain base subunit 2
SSE: splice site effect
TAG: triacylglycerol
VEP: variant effect predictor
VPS13C: vacuolar protein sorting 13 homolog C
YIF1A: Yip1 interacting factor homolog A
