# Supplementary material for: Exploratory identification of coding and splicing-related SNV variants in A1A1 and A2A2 β-casein Holstein dairy cows
Source: J Anim Sci. 2026 Jul 29;104:skag233. doi: 10.1093/jas/skag233 (PMC13461648; doi:10.1093/jas/skag233)
Supplement: Supplementary file 1 [file skag233_supplementary_data.docx]

**Exploratory identification of coding and splicing-related SNV variants in A1A1 and A2A2 β-casein Holstein dairy cows**

**^1^Lucía Jiménez-Montenegro*, ^2^Olaia Urrutia*, ^1^Ángela Cánovas*.**

^1^Centre for Genetic Improvement of Livestock, Department of Animal Biosciences, University of Guelph, N1G 2W1, Guelph, ON, Canada.

^2^Institute on Innovation and Sustainable Development in Food Chain (IS-FOOD), Public University of Navarre (UPNA), Campus de Arrosadia, 31006, Pamplona, Spain.

*Correspondence: olaia.urrutia@unavarra.es (O.U.); acanovas@uoguelph.ca (A.C.)

**Supplementary Table S1.**  Main single nucleotide variants (SNVs) predicted to lead amino acid change (AAC) (SIFT < 0.05) detected in expressed transcripts of A1A1 β-casein group of cows (0.2 FPKM).

| **A1A1 associated SNV ID** | **Transcript** | **Gene** | **Consequence** | **SIFT^1^** | **A1A1 average FPKM^2^** | **A2A2 average FPKM^2^** | **FDR^3^** | **Main molecular Function** |
| --- | --- | --- | --- | --- | --- | --- | --- | --- |
| **Mitochondrial oxidative phosphorylation (OXPHOS) and electron transport chain (ETC)** | | | | | | | | |
| 24_41416489 G/A | NDUFV2-203 | *NDUFV2* | missense variant | 0.01 | 1.937 | 5.868 | 0.643 | ETC Complex I, NADH dehydrogenase |
| 25 21261249 G/A | NDUFAB1-201 | *NDUFAB1* | missense variant | 0.04 | 35.105 | 36.775 | 0.992 |  |
| 9_14835368_T/G | COX7A2-201 | *COX7A2* | missense variant | 0.02 | 58.850 | 68.741 | 0.997 | ETC Complex IV, cytochrome c oxidase |
| 9_14835368_T/G | COX7A2-202 | *COX7A2* | missense variant | 0.01 | 0.257 | 0.177 | 0.959 |  |
| 1_10691073_T/A | ATP5PF-201 | *ATP5PF* | missense variant | 0 | 1.869 | 2.091 | 0.999 | ATP synthase subunit (Complex V) |
| **Mitochondrial integrity and function** | | | | | | | | |
| 17_70324754_A/G | PISD-205 | *PISD* | missense variant | 0.01 | 0.503 | 0.094 | 0.470 | Mitochondrial phospholipid synthesis |
| 11_48689263_C/T | IMMT-203 | *IMMT* | missense variant | 0 | 1.939 | 1.289 | 0.909 | Structure of the inner membrane of mitochondria |
| 11_48714033_G/T | IMMT-203 | *IMMT* | missense variant, splice-region variant | 0.01 | 1.939 | 1.289 | 0.909 |  |
| **Protein synthesis** | | | | | | | | |
| **13_7321761_C/T** | **ESF1-201** | ***ESF1*** | **missense variant** | **0.03** | **0.225** | **0.000** | **0.032** | Ribosome biogenesis |
| **Lipid metabolism** | | | | | | | | |
| 27_15192468_C/T | ACSL1-206 | *ACSL1* | missense variant, splice-region variant | 0.02 | 1.563 | 0.446 | 0.608 | Fatty Acid activation |
| 27_15192468_C/T | ACSL1-204 | *ACSL1* | missense variant, splice-region variant | 0.02 | 6.171 | 7.026 | 0.999 |  |
| 27_15192468_C/T | ACSL1-203 | *ACSL1* | missense variant, splice-region variant | 0.02 | 0.207 | 0.092 | 0.881 |  |
| 27_15192468_C/T | ACSL1-202 | *ACSL1* | missense variant, splice-region variant | 0.02 | 0.805 | 0.475 | 0.870 |  |
| 27_15192468_C/T | ACSL1-205 | *ACSL1* | missense variant, splice-region variant | 0.02 | 1.041 | 0.328 | 0.789 |  |
| 21_2797002_G/C | ATP10A-201 | *ATP10A* | missense variant | 0 | 0.006 | 0.214 | 0.069 | Phospholipid flippase |
| **Peroxisomal metabolism** | | | | | | | | |
| 9_74805010_C/A | PEX7-201 | *PEX7* | missense variant | 0 | 0.457 | 0.041 | 0.201 | Peroxisomal enzyme import |
| 9_74833653_G/A | PEX7-204 | *PEX7* | missense variant | 0.02 | 2.068 | 2.917 | 0.983 |  |
| 9_74833653_G/A | PEX7-201 | *PEX7* | missense variant | 0.05 | 0.457 | 0.041 | 0.201 |  |
| **Autophagy/mitophagy** | | | | | | | | |
| 17_69083107_G/A | MTMR3-204 | *MTMR3* | missense variant | 0.01 | 0.360 | 0.814 | 0.876 | Negative regulator of autophagy |
| **Splicing Regulation** | | | | | | | | |
| 1_1979297_G/A | SON-201 | *SON* | missense variant | 0.02 | 2.312 | 6.623 | 0.081 | Splicing of essential transcripts |
| 1_1979297_G/A | SON-205 | *SON* | missense variant | 0.01 | 1.046 | 1.360 | 0.980 |  |
| **1_1979297_G/A** | **SON-206** | ***SON*** | **missense variant** | **0.01** | **0.217** | **0.000** | **0.012** |  |
| 25_2291571_G/A | SRRM2-201 | *SRRM2* | missense variant | 0 | 2.83 | 3.06 | 0.999 | Splicing factor and a core component of nuclear speckles. |
| 25_2291571_G/A | SRRM2-202 | *SRRM2* | missense variant | 0 | 0.384 | 0.256 | 0.947 |  |
| 25_2291571_G/A | SRRM2-203 | *SRRM2* | missense variant | 0 | 0.693 | 1.055 | 0.970 |  |
| 25_2291571_G/A | SRRM2-204 | *SRRM2* | missense variant | 0 | 0.545 | 0.503 | 0.992 |  |
| **Cellular signalling and apoptosis** | | | | | | | | |
| **21_69229824_G/A** | **SIVA1-201** | ***SIVA1*** | **missense variant** | **0** | **0.000** | **0.582** | **0.046** | Pro-apoptotic signalling |
| 23_32397444_T/C | CARMIL1-201 | *CARMIL1* | missense variant, splice-region variant | 0 | 0.636 | 1.299 | 0.840 | Actin cytoskeleton dynamics |
| **23_32397444_T/C** | **CARMIL1-203** | ***CARMIL1*** | **missense variant, splice-region variant** | **0** | **0.012** | **0.630** | **0.019** |  |
| 23_32397444_T/C | CARMIL1-204 | *CARMIL1* | missense variant, splice-region variant | 0 | 6.218 | 8.199 | 0.973 |  |

Abbreviations: *NDUFV2* = NADH dehydrogenase [ubiquinone] flavoprotein 2; *NDUFAB1* = NADH dehydrogenase [ubiquinone] 1 alpha subcomplex assembly factor 1; *COX7A2* = Cytochrome c oxidase subunit 7A2; *ATP5PF* = ATP synthase peripheral stalk subunit F; *PISD* = Phosphatidylserine decarboxylase; *IMMT* = Inner mitochondrial membrane protein (Mitofilin); *ESF1* = ESF1 ribosome biogenesis factor; *ACSL1* = Acyl-CoA synthetase long-chain family member 1; *ATP10A* = ATPase phospholipid transporting 10A; *PEX7* = Peroxisomal biogenesis factor 7; *MTMR3* = Myotubularin related protein 3; *SON* = SON DNA and RNA binding protein;; *SIVA1* = SIVA apoptosis inducing factor 1; *CARMIL1* = Capping protein regulator and myosin 1 linker 1.

^1^SIFT (Sorting Intolerant from Tolerant) predicts the functional impact of amino acid substitutions; variants with SIFT < 0.05 indicates a predicted functional impact and consequences on transcripts.

^2^Gene expression is given in FPKM (fragments per kilobase per million mapped reads), calculated to normalize read counts per library size and gene length.

^3^FDR = False Discovery Rate. Differentially expressed (DE) mRNA isoforms are indicated in bold (FDR < 0.05).

**Supplementary Table S2.** Main single nucleotide variants (SNVs) predicted to lead amino acid change (AAC) (SIFT < 0.05) detected in expressed transcripts of A2A2 β-casein group of cows (≥ 0.2 FPKM).

| **A2A2 associated SNV ID** | **Transcript** | **Gene** | **Consequence** | **SIFT^1^** | **A1A1 average FPKM^2^** | **A2A2 average FPKM^2^** | **FDR^3^** | **Main Molecular Function** |
| --- | --- | --- | --- | --- | --- | --- | --- | --- |
| **Mitochondrial integrity and function** | | | | | | | | |
| 1_73849107_G/C | OPA1-201 | *OPA1* | missense variant | 0.02 | 0.941 | 0.564 | 0.949 | Mitochondrial morphology and cristae organisation |
| 1_73849107_G/C | OPA1-204 | *OPA1* | missense variant | 0.02 | 0.279 | 0.447 | 0.981 |  |
| 1_73851821_C/T | OPA1-208 | *OPA1* | missense variant | 0 | 4.374 | 5.237 | 0.991 |  |
| 1_73851821_C/T | OPA1-201 | *OPA1* | missense variant | 0 | 0.941 | 0.564 | 0.949 |  |
| 1_73851821_C/T | OPA1-206 | *OPA1* | missense variant | 0 | 0.113 | 0.285 | 0.934 |  |
| 1_73851821_C/T | OPA1-204 | *OPA1* | missense variant | 0 | 0.279 | 0.447 | 0.981 |  |
| 10_48144355_C/T | VPS13C-203 | *VPS13C* | missense variant | 0.01 | 0.271 | 0.367 | 0.986 | Mitochondrial quality control |
| 17_62634248_G/T | SIRT4-201 | *SIRT4* | missense variant | 0.03 | 0.072 | 0.239 | 0.750 | Mitochondrial metabolic regulation |
| **Protein synthesis** | | | | | | | | |
| 1_106184720_T/C | NMD3-201 | *NMD3* | missense variant | 0.03 | 0.428 | 1.177 | 0.879 | Ribosomal Biogenesis |
| **Protein turnover** | | | | | | | | |
| 10_76680000_T/C | HSPA2-203 | *HSPA2* | missense variant | 0 | 0.148 | 0.276 | 0.963 | Basal protein folding and proteostasis |
| 23_27525598_A/G | HSPA1L-204 | *HSPA1L* | missense variant | 0 | 2.677 | 3.939 | 0.888 | Stress-responsive protein folding and proteostasis |
| 23_27525598_A/G | HSPA1L-201 | *HSPA1L* | missense variant | 0 | 0.230 | 0.209 | 0.995 |  |
| **Lipid Metabolism** |  |  |  |  |  |  |  |  |
| 11_86097586_C/T | LPIN1-201 | *LPIN1* | missense variant | 0 | 22.079 | 30.012 | 0.902 | Triacylglycerol synthesis. |
| 19_50791479_C/T | FASN-202 | *FASN* | missense variant | 0 | 6.080 | 6.755 | 0.995 | *De novo* fatty acid synthesis |
| 10_88784402_C/T | SPTLC2-201 | *SPTLC2* | missense variant | 0.02 | 0.578 | 0.330 | 0.914 | Sphingolipid biosynthesis |
| 1_84188257_G/A | ATP11B-208 | *ATP11B* | missense variant | 0 | 0.174 | 0.227 | 0.997 | Phospholipid flippase |
| **Vesicle trafficking** | | | | | | | | |
| 6_97694187_T/C | SEC31A-206 | *SEC31A* | missense variant | 0 | 0.724 | 0.566 | 0.977 | ER-to-Golgi transport |
| 6_97694187_T/C | SEC31A-201 | *SEC31A* | missense variant | 0.01 | 0.435 | 0.790 | 0.923 |  |
| 6_97694187_T/C | SEC31A-203 | *SEC31A* | missense variant | 0 | 1.097 | 0.934 | 0.964 |  |
| 6_97694187_T/C | SEC31A-205 | *SEC31A* | missense variant | 0.02 | 0.259 | 0.101 | 0.785 |  |
| 5_40643662_A/G | LRRK2-201 | *LRRK2* | missense variant | 0 | 0.069 | 0.371 | 0.468 | Vesicle trafficking regulation |
| 5_40646293_C/T | LRRK2-201 | *LRRK2* | missense variant, splice-region variant | 0.01 | 0.069 | 0.371 | 0.468 |  |
| 22_380933_A/G | DBNL-204 | *DBNL* | missense variant, splice-region variant | 0.04 | 10.966 | 15.438 | 0.978 | Actin-dependent endocytosis |
| **Cellular signalling** | | | | | | | | |
| 20_35421065_C/G | RICTOR-205 | *RICTOR* | missense variant, splice-region variant | 0.01 | 0.202 | 0.217 | 0.997 | mTORC2 signalling and metabolic regulation |
| 20_35421069_T/A | RICTOR-205 | *RICTOR* | missense variant | 0 | 0.202 | 0.217 | 0.997 |  |
| **RNA processing** | | | | | | | | |
| 21_29153940_C/T | SNRPA1-204 | *SNRPA1* | missense variant | 0 | 0.249 | 0.576 | 0.904 | Spliceosome component required for pre-mRNA splicing |
| 21_29153970_C/T | SNRPA1-204 | *SNRPA1* | missense variant | 0 | 0.249 | 0.576 | 0.904 |  |
| 21_29153973_G/A | SNRPA1-204 | *SNRPA1* | missense variant | 0 | 0.249 | 0.576 | 0.904 |  |

Abbreviations: *OPA1* = Optic atrophy 1 (mitochondrial dynamin-like GTPase); *VPS13C* = Vacuolar protein sorting 13 homolog C; *SIRT4* = Sirtuin 4 (mitochondrial); *NMD3* = Nuclear export adaptor protein NMD3; *HSPA2* = Heat shock protein family A (Hsp70) member 2; *HSPA1L* = Heat shock protein family A (Hsp70) member 1-like; *FASN* = Fatty acid synthase; *SPTLC2* = Serine palmitoyltransferase long chain base subunit 2; *ATP11B* = ATPase phospholipid transporting 11B; *SEC31A* = SEC31 homolog A, COPII coat complex component; *LRRK2* = Leucine rich repeat kinase 2; *DBNL* = Drebrin like protein; *RICTOR* = RPTOR independent companion of MTOR complex 2.

^1^SIFT (Sorting Intolerant from Tolerant) predicts the functional impact of amino acid substitutions; variants with SIFT < 0.05 indicates a predicted functional impact and consequences on transcripts.

^2^Gene expression is given in FPKM (fragments per kilobase per million mapped reads), calculated to normalize read counts per library size and gene length.

^3^FDR = False Discovery Rate. Differentially expressed (DE) mRNA isoforms are indicated in bold (FDR < 0.05).

**Supplementary Table S3.** Theoretical VEP annotations for non-expressed transcripts sharing genomic positions with SNVs predicted to cause amino acid changes (AAC) in expressed transcripts of A1A1 β-casein cows.

| **A1A1 associated SNV ID** | **Transcript** | **Gene** | **Consequence** | **SIFT^1^** | **A1A1 average FPKM^2^** | **A2A2 average FPKM^2^** | **FDR^3^** | **Main molecular Function** |
| --- | --- | --- | --- | --- | --- | --- | --- | --- |
| **Mitochondrial oxidative phosphorylation (OXPHOS) and electron transport chain (ETC)** | | | | | | | | |
| 29_45563226_G/A | NDUFS8-201 | *NDUFS8* | missense variant | 0.02 | 0.114 | 0.000 | 0.106 | ETC Complex I, NADH dehydrogenase |
| **Mitochondrial integrity and function** | | | | | | | | |
| 3_14592319_C/G | SLC25A44-203 | *SLC25A44* | missense variant | 0 | 0.098 | 0.066 | 0.987 | Mitochondrial amino acid transport |
| 3_14592319_C/G | SLC25A44-201 | *SLC25A44* | missense variant | 0 | 0.041 | 0.000 | 0.118 |  |
| **25_36978649_A/G** | **PTCD1-202** | ***PTCD1*** | **missense variant** | **0.01** | **0.126** | **0.000** | **0.048** | Mitochondrial mRNA stability and translation |
| 17_70324754_A/G | PISD-204 | *PISD* | missense variant | 0 | 0.000 | 0.000 | - | Mitochondrial phospholipid synthesis |
| 17_70324754_A/G | PISD-201 | *PISD* | missense variant | 0 | 0.000 | 0.000 | - |  |
| 17_70324754_A/G | PISD-208 | *PISD* | missense variant | 0 | 0.000 | 0.000 | - |  |
| 17_70324754_A/G | PISD-202 | *PISD* | missense variant | 0 | 0.102 | 0.000 | 0.067 |  |
| 17_70324754_A/G | PISD-203 | *PISD* | missense variant | 0 | 0.000 | 0.000 | - |  |
| 17_70324754_A/G | PISD-206 | *PISD* | missense variant | 0 | 0.000 | 0.000 | - |  |
| **Protein synthesis** | | | | | | | | |
| 13_7321761_C/T | ESF1-202 | *ESF1* | missense variant | 0.03 | 0.000 | 0.000 | - |  |
| **Lipid metabolism** | | | | | | | | |
| 27_15192468_C/T | ACSL1-201 | *ACSL1* | missense variant, splice-region variant | 0.02 | 0.000 | 0.000 | - | Fatty Acid activation |
| 27_15192468_C/T | ACSL1-208 | *ACSL1* | missense variant, splice-region variant | 0.02 | 0.198 | 0.198 | 0.995 |  |
| 27_15192468_C/T | ACSL1-209 | *ACSL1* | missense variant, splice-region variant | 0.02 | 0.000 | 0.000 | - |  |
| 21_2797002_G/C | ATP10A-202 | *ATP10A* | missense variant | 0 | 0.179 | 0.187 | 0.998 |  |
| **Peroxisomal metabolism** | | | | | | | | |
| 9_74833653_G/A | PEX7-206 | *PEX7* | missense variant | 0.03 | 0.000 | 0.000 | - | Peroxisomal enzyme import |
| 9_74833653_G/A | PEX7-202 | *PEX7* | missense variant | 0.04 | 0.000 | 0.026 | - |  |
| 9_74833653_G/A | PEX7-205 | *PEX7* | missense variant | 0.02 | 0.000 | 0.000 | - |  |
| 9_74833653_G/A | PEX7-203 | *PEX7* | missense variant | 0.04 | 0.000 | 0.000 | - |  |
| **Autophagy/mitophagy** | | | | | | | | |
| 9_43675027_C/T | ATG5-205 | *ATG5* | missense variant | 0.05 | 0.143 | 0.061 | 0.771 | Autophagosome formation |
| 9_43675027_C/T | ATG5-203 | *ATG5* | missense variant | 0.03 | 0.000 | 0.136 | 0.252 |  |
| 17_69083107_G/A | MTMR3-202 | *MTMR3* | missense variant | 0.01 | 0.008 | 0.003 | - | Negative regulator of autophagy |
| 17_69083107_G/A | MTMR3-206 | *MTMR3* | missense variant | 0.02 | 0.123 | 0.000 | 0.029 |  |
| 17_69083107_G/A | MTMR3-205 | *MTMR3* | missense variant | 0.02 | 0.126 | 0.882 | 0.173 |  |
| **Splicing Regulation** | | | | | | | | |
| 1_1979297_G/A | SON-204 | *SON* | missense variant | 0.01 | 0.017 | 0.014 | 0.996 | Splicing of essential transcripts |
| 1_1979297_G/A | SON-203 | *SON* | missense variant | 0.01 | 0.000 | 0.000 | - |  |
| **Cellular stress, apoptosis and signalling** | | | | | | | | |
| 17_57793695_G/C | NOS1-203 | *NOS1* | missense variant | 0.02 | 0.030 | 0.014 | 0.889 | Nitric oxide signalling |
| 17_57793695_G/C | NOS1-201 | *NOS1* | missense variant | 0.01 | 0.000 | 0.000 | - |  |
| 23_32397444_T/C | CARMIL1-202 | *CARMIL1* | missense variant, splice-region variant | 0 | 0.010 | 0.066 | 0.558 | Actin cytoskeleton dynamics |
| 23_32397444_T/C | CARMIL1-205 | *CARMIL1* | missense variant, splice-region variant | 0 | 0.033 | 0.188 | 0.572 |  |
| 23_32397444_T/C | CARMIL1-206 | *CARMIL1* | missense variant, splice-region variant | 0 | 0.084 | 0.131 | 0.986 |  |

Abbreviations: *NDUFS8* = NADH dehydrogenase [ubiquinone] iron-sulfur protein 8; *SLC25A44* = Solute carrier family 25 member 44; *PTCD1* = Pentatricopeptide repeat domain protein 1; *PISD* = Phosphatidylserine decarboxylase; *ESF1* = ESF1 ribosome biogenesis factor; *ACSL1* = Acyl-CoA synthetase long-chain family member 1; *ATP10A* = ATPase phospholipid transporting 10A; *PEX7* = Peroxisomal biogenesis factor 7; *ATG5* = Autophagy related 5; *MTMR3* = Myotubularin related protein 3; *SON* = SON DNA and RNA binding protein; *NOS1* = Nitric oxide synthase 1; *CARMIL1* = Capping protein regulator and myosin 1 linker 1.

^1^SIFT (Sorting Intolerant from Tolerant) predicts the functional impact of amino acid substitutions; variants with SIFT < 0.05 indicates a predicted functional impact on transcripts.

^2^Gene expression is given in FPKM (fragments per kilobase per million mapped reads), calculated to normalize read counts per library size and gene length.

^3^FDR = False Discovery Rate. Differentially expressed (DE) mRNA isoforms are indicated in bold (FDR < 0.05) and (–) indicates not reported due to low or absent expression.

**Supplementary Table S4.** Theoretical VEP annotations for non-expressed transcripts sharing genomic positions with SNVs predicted to cause amino acid changes (AAC) in expressed transcripts of A2A2 β-casein cows.

| **A2A2 associated SNV ID** | **Transcript** | **Gene** | **Consequence** | **SIFT^1^** | **A1A1 average FPKM^2^** | **A2A2 average FPKM^2^** | **FDR^3^** | **Main Molecular Function** |
| --- | --- | --- | --- | --- | --- | --- | --- | --- |
| **Mitochondrial integrity and function** | | | | | | | | |
| 1_73849107_G/C | OPA1-207 | *OPA1* | missense variant | 0.03 | 0.021 | 0.000 | - | Mitochondrial morphology and cristae organisation |
| 1_73849107_G/C | OPA1-205 | *OPA1* | missense variant | 0.02 | 0.000 | 0.079 | 0.100 |  |
| 1_73851821_C/T | OPA1-207 | *OPA1* | missense variant | 0 | 0.021 | 0.000 | - |  |
| 1_73851821_C/T | OPA1-205 | *OPA1* | missense variant | 0 | 0.000 | 0.079 | 0.100 |  |
| 1_73851821_C/T | OPA1-203 | *OPA1* | missense variant | 0 | 0.000 | 0.000 | - |  |
| 1_73851821_C/T | OPA1-209 | *OPA1* | missense variant | 0 | 0.000 | 0.000 | - |  |
| 7_109738303_G/A | SLC25A46-202 | *SLC25A46* | missense variant | 0 | 0.000 | 0.000 | - | Mitochondrial membrane organisation |
| 7_109738303_G/A | SLC25A46-203 | *SLC25A46* | missense variant | 0 | 0.000 | 0.002 | - |  |
| 10_48144355_C/T | VPS13C-202 | *VPS13C* | missense variant | 0.01 | 0.000 | 0.000 | - | Mitochondrial quality control |
| 10_48144355_C/T | VPS13C-201 | *VPS13C* | missense variant | 0.01 | 0.000 | 0.011 | 0.197 |  |
| 17_62634248_G/T | SIRT4-203 | *SIRT4* | missense variant | 0.02 | 0.000 | 0.000 | - | Mitochondrial metabolic regulation |
| 17_62634248_G/T | SIRT4-202 | *SIRT4* | missense variant | 0.02 | 0.109 | 0.131 | 0.997 |  |
| **Protein synthesis** | | | | | | | | |
| 1_106184720_T/C | NMD3-202 | *NMD3* | missense variant | 0.03 | 0.137 | 0.047 | 0.800 | 60 S Ribosomal subunit export |
| **Proteostasis and protein turnover** | | | | | | | | |
| 10_76680000_T/C | HSPA2-202 | *HSPA2* | missense variant | 0 | 0.006 | 0.065 | 0.249 | Basal protein folding and proteostasis |
| 10_76680000_T/C | HSPA2-201 | *HSPA2* | missense variant | 0 | 0.000 | 0.000 | - |  |
| 10_76680000_T/C | HSPA2-204 | *HSPA2* | missense variant | 0 | 0.025 | 0.000 | 0.085 |  |
| 23_27525598_A/G | HSPA1L-206 | *HSPA1L* | missense variant | 0 | 0.000 | 0.000 | - | Stress-responsive protein folding and proteostasis |
| 23_27525598_A/G | HSPA1L-202 | *HSPA1L* | missense variant | 0 | 0.038 | 0.011 | 0.755 |  |
| 23_27525598_A/G | HSPA1L-205 | *HSPA1L* | missense variant | 0 | 0.041 | 0.008 | 0.379 |  |
| 23_27525598_A/G | HSPA1L-203 | *HSPA1L* | missense variant | 0 | 0.000 | 0.007 | - |  |
| 23_27525598_A/G | HSPA1L-207 | *HSPA1L* | missense variant | 0 | 0.042 | 0.000 | 0.102 |  |
| 23_27525598_A/G | HSPA1L-208 | *HSPA1L* | missense variant | 0 | 0.006 | 0.031 | 0.537 |  |
| 10_88494420_C/T | POMT2-201 | *POMT2* | missense variant | 0.02 | 0.006 | 0.000 | - | Post-translational modifications RE enzyme |
| 10_88494420_C/T | POMT2-205 | *POMT2* | missense variant | 0.05 | 0.000 | 0.000 | - |  |
| 10_88494420_C/T | POMT2-207 | *POMT2* | missense variant | 0.02 | 0.000 | 0.000 | - |  |
| 10_88494420_C/T | POMT2-204 | *POMT2* | missense variant | 0.01 | 0.089 | 0.113 | 0.989 |  |
| 10_88494420_C/T | POMT2-202 | *POMT2* | missense variant | 0.01 | 0.098 | 0.027 | 0.595 |  |
| 10_88494420_C/T | POMT2-206 | *POMT2* | missense variant | 0.01 | 0.000 | 0.012 | - |  |
| **Lipid metabolism** | | | | | | | | |
| 11_86097586_C/T | LPIN1-205 | *LPIN1* | missense variant | 0 | 0.019 | 0.036 | 0.966 | Triacylglycerol synthesis. |
| 11_86097586_C/T | LPIN1-203 | *LPIN1* | missense variant | 0 | 0.000 | 0.000 | - |  |
| 11_86097586_C/T | LPIN1-204 | *LPIN1* | missense variant | 0 | 0.000 | 0.000 | - |  |
| 11_86097586_C/T | LPIN1-202 | *LPIN1* | missense variant | 0 | 0.066 | 0.026 | 0.673 |  |
| 5_119891935_G/A | CPT1B-202 | *CPT1B* | missense variant | 0 | 0.185 | 0.020 | 0.230 | Mitochondrial fatty acid import |
| 5_119891935_G/A | CPT1B-203 | *CPT1B* | missense variant | 0 | 0.115 | 0.173 | 0.982 |  |
| 10_88784402_C/T | SPTLC2-202 | *SPTLC2* | missense variant | 0.02 | 0.050 | 0.066 | 0.989 | Sphingolipid biosynthesis |
| 1_84188257_G/A | ATP11B-209 | *ATP11B* | missense variant | 0 | 0.000 | 0.000 | - | Phospholipid flippase |
| 1_84188257_G/A | ATP11B-203 | *ATP11B* | missense variant | 0 | 0.105 | 0.011 | 0.206 |  |
| 1_84188257_G/A | ATP11B-202 | *ATP11B* | missense variant | 0 | 0.000 | 0.000 | - |  |
| 1_84188257_G/A | ATP11B-210 | *ATP11B* | missense variant | 0 | 0.000 | 0.000 | - |  |
| 1_84188257_G/A | ATP11B-205 | *ATP11B* | missense variant | 0 | 0.000 | 0.000 | - |  |
| 1_84188257_G/A | ATP11B-201 | *ATP11B* | missense variant | 0 | 0.000 | 0.000 | - |  |
| 1_84188257_G/A | ATP11B-204 | *ATP11B* | missense variant | 0 | 0.000 | 0.000 | - |  |
| 1_84188257_G/A | ATP11B-207 | *ATP11B* | missense variant | 0 | 0.000 | 0.037 | 0.171 |  |
| 1_84188257_G/A | ATP11B-206 | *ATP11B* | missense variant | 0 | 0.000 | 0.027 | 0.201 |  |
| **Membrane organisation and vesicle trafficking** | | | | | | | | |
| 11_106068121_G/C | ABCA2-203 | *ABCA2* | missense variant | 0.01 | 0.047 | 0.085 | 0.964 | Intracellular lipid transport |
| 19_61342103_A/T | ABCA5-202 | *ABCA5* | missense variant | 0.01 | 0.071 | 0.050 | 0.961 | Lipid transport and lysosomal function |
| 19_61342103_A/T | ABCA5-201 | *ABCA5* | missense variant | 0.01 | 0.136 | 0.023 | 0.297 |  |
| 6_97694187_T/C | SEC31A-202 | *SEC31A* | missense variant | 0 | 0.000 | 0.041 | 0.140 | ER-to-Golgi transport |
| 6_97694187_T/C | SEC31A-204 | *SEC31A* | missense variant | 0 | 0.025 | 0.052 | 0.920 |  |
| 6_97694187_T/C | SEC31A-201 | *SEC31A* | missense variant | 0.01 | 0.435 | 0.790 | 0.923 |  |
| 6_97694187_T/C | SEC31A-203 | *SEC31A* | missense variant | 0 | 1.097 | 0.934 | 0.964 |  |
| 6_97694187_T/C | SEC31A-205 | *SEC31A* | missense variant | 0.02 | 0.259 | 0.101 | 0.785 |  |
| 11_97303380_A/C | MVB12B-208 | *MVB12B* | missense variant | 0 | 0.033 | 0.154 | 0.636 | Multivesicular bodies formation |
| 11_97303380_A/C | MVB12B-207 | *MVB12B* | missense variant | 0 | 0.124 | 0.000 | 0.109 |  |
| **5_40643662_A/G** | **LRRK2-202** | ***LRRK2*** | **missense variant** | **0.01** | **0.117** | **0.000** | **0.026** | Vesicle trafficking regulation |
| **Cellular stress, apoptosis and signalling** | | | | | | | | |
| 17_57793559_C/G | NOS1-203 | *NOS1* | missense variant | 0.03 | 0.030 | 0.014 | 0.889 | Nitric oxide synthesis and signalling |
| 17_57793559_C/G | NOS1-201 | *NOS1* | missense variant | 0.05 | 0.000 | 0.000 | - |  |
| **20_35421065_C/G** | **RICTOR-204** | ***RICTOR*** | **missense variant, splice-region variant** | **0.01** | **0.000** | **0.085** | **0.045** | mTORC2 signalling and metabolic regulation. |
| **20_35421065_C/G** | **RICTOR-206** | ***RICTOR*** | **missense variant, splice-region variant** | **0.01** | **0.022** | **0.000** | **0.087** |  |
| 20_35421065_C/G | RICTOR-207 | *RICTOR* | missense variant, splice-region variant | 0.01 | 0.027 | 0.083 | 0.831 |  |
| 20_35421065_C/G | RICTOR-201 | *RICTOR* | missense variant, splice-region variant | 0.01 | 0.000 | 0.000 | - |  |
| **20_35421069_T/A** | **RICTOR-204** | ***RICTOR*** | **missense variant** | **0** | **0.000** | **0.085** | **0.045** |  |
| 20_35421069_T/A | RICTOR-206 | *RICTOR* | missense variant | 0 | 0.022 | 0.000 | 0.087 |  |
| 20_35421069_T/A | RICTOR-207 | *RICTOR* | missense variant | 0 | 0.027 | 0.083 | 0.831 |  |
| 20_35421069_T/A | RICTOR-201 | *RICTOR* | missense variant | 0 | 0.000 | 0.000 | - |  |
| **Other** | | | | | | | | |
| 11_98056770_G/T | GARNL3-209 | *GARNL3* | missense variant, splice-region variant | 0 | 0.000 | 0.000 | - | Regulation of small GTPases. |
| 11_98056770_G/T | GARNL3-208 | *GARNL3* | missense variant, splice-region variant | 0 | 0.000 | 0.000 | - |  |
| 11_98056770_G/T | GARNL3-204 | *GARNL3* | missense variant, splice-region variant | 0 | 0.000 | 0.000 | - |  |
| **11_98056770_G/T** | **GARNL3-207** | ***GARNL3*** | **missense variant, splice-region variant** | **0** | **0.183** | **0.000** | **0.035** |  |
| 11_98056770_G/T | GARNL3-203 | *GARNL3* | missense variant, splice-region variant | 0 | 0.040 | 0.139 | 0.834 |  |

Abbreviations: *OPA1* = Optic atrophy 1 (mitochondrial dynamin-like GTPase); *SLC25A46* = Solute carrier family 25 member 46; *VPS13C* = Vacuolar protein sorting 13 homolog C; *SIRT4* = Sirtuin 4 (mitochondrial); *NMD3* = Nuclear export adaptor protein NMD3; *HSPA2* = Heat shock protein family A (Hsp70) member 2; *HSPA1L* = Heat shock protein family A (Hsp70) member 1-like; *POMT2* = Protein O-mannosyltransferase 2; *LPIN1* = Lipin 1; *CPT1B* = Carnitine palmitoyltransferase 1B; *SPTLC2* = Serine palmitoyltransferase long chain base subunit 2; *ATP11B* = ATPase phospholipid transporting 11B; *ABCA2* = ATP binding cassette subfamily A member 2; *ABCA5* = ATP binding cassette subfamily A member 5; *SEC31A* = SEC31 homolog A, COPII coat complex component; *MVB12B* = Multivesicular body subunit 12B; *LRRK2* = Leucine rich repeat kinase 2; *NOS1* = Nitric oxide synthase 1; *RICTOR* = RPTOR independent companion of MTOR complex 2; *GARNL3* = GTPase activating Rap/RanGAP domain like 3.

^1^SIFT (Sorting Intolerant from Tolerant) predicts the functional impact of amino acid substitutions; variants with SIFT < 0.05 indicates a predicted functional impact and consequences on transcripts.

^2^Gene expression is given in FPKM (fragments per kilobase per million mapped reads), calculated to normalize read counts per library size and gene length.

^3^FDR = False Discovery Rate. Differentially expressed (DE) mRNA isoforms are indicated in bold (FDR < 0.05) and (–) indicates not reported due to low or absent expression.

**Supplementary Table S5.** Main single nucleotide variants (SNVs) leading to splice site effect (SSE) located in genes with differentially expressed (DE) mRNA isoforms in A1A1 β-casein group of cows.

| **A1A1 associated SNV ID** | **Transcript** | **Gene** | **Consequence** | **Impact** | **A1A1 Average FPKM^2^** | **A2A2 Average FPKM^2^** | **FDR^3^** | **Main Molecular Function** |
| --- | --- | --- | --- | --- | --- | --- | --- | --- |
| **Mitochondrial redox balance and cell survival** | | | | | | | | |
| - | AIFM2_3^1^ | *AIFM2* | - | - | 0.306 | 0.051 | 0.457 | Mitochondrial redox control and ferroptosis inhibition |
| **-** | **AIFM2_4^1^** | ***AIFM2*** | **-** |  | **0.006** | **0.291** | **0.034** |  |
| - | AIFM2_8^1^ | *AIFM2* | - | - | 0.409 | 0.416 | 0.998 |  |
| - | AIFM2_9^1^ | *AIFM2* | - | - | 1.488 | 0.362 | 0.370 |  |
| 28_26347419_A/G | AIFM2-201 | *AIFM2* | splice-donor variant | HIGH | 0.413 | 0.983 | 0.947 |  |
| **Cytoskeleton organisation and secretion mechanics** | | | | | | | | |
| - | CARMIL1_1 | *CARMIL1* | - | - | 2.565 | 5.277 | 0.890 | Actin cytoskeleton dynamics |
| **-** | **CARMIL1_2** | ***CARMIL1*** | **-** | **-** | **0.000** | **0.849** | **0.034** |  |
| - | CARMIL1_3 | *CARMIL1* | - | - | 7.140 | 9.327 | 0.984 |  |
| - | CARMIL1_4 | *CARMIL1* | - | - | 6.281 | 12.682 | 0.446 |  |
| - | CARMIL1_5^1^ | *CARMIL1* | - | - | 0.964 | 0.985 | 0.998 |  |
| - | CARMIL1_6^1^ | *CARMIL1* | - | - | 0.000 | 0.345 | 0.134 |  |
| 23_32397444_T/C | CARMIL1-201 | *CARMIL1* | missense variant, splice-region variant | MODERATE/SIFT 0 | 0.636 | 1.299 | 0.840 |  |
| **23_32397444_T/C** | **CARMIL1-203** | ***CARMIL1*** | **missense variant, splice-region variant** | **MODERATE/SIFT 0** | **0.012** | **0.630** | **0.019** |  |
| 23_32397444_T/C | CARMIL1-204 | *CARMIL1* | missense variant, splice-region variant | MODERATE/SIFT 0 | 6.218 | 8.199 | 0.973 |  |
| **Milk fat globule (MFG) structure and secretion** | | | | | | | | |
| - | MFGE8_1 | *MFGE8* | - | - | 31.666 | 61.910 | 0.793 | Milk fat globule membrane (MFGM) component |
| - | MFGE8_2^1^ | *MFGE8* | - | - | 23.905 | 26.594 | 0.998 |  |
| **-** | **MFGE8_3^1^** | ***MFGE8*** | **-** | **-** | **0.493** | **8.297** | **0.003** |  |
| - | MFGE8_4 | *MFGE8* | - | - | 0.050 | 0.036 | - |  |
| 21_20476454_C/T | MFGE8-201 | *MFGE8* | splice-donor variant | HIGH | 0.650 | 0.422 | 0.972 |  |
| 21_20476454_C/T | MFGE8-204 | *MFGE8* | splice-donor variant | HIGH | 124.898 | 175.413 | 0.907 |  |
| **Vesicular trafficking** | | | | | | | | |
| - | YIF1A_2 | *YIF1A* | - | - | 3.094 | 11.596 | 0.386 | Golgi vesicular trafficking |
| - | YIF1A_3 | *YIF1A* | - | - | 0.670 | 0.977 | 0.982 |  |
| **-** | **YIF1A_4** | ***YIF1A*** | **-** | **-** | **0.405** | **0.000** | **0.048** |  |
| 29_44342558_C/G | YIF1A-201 | *YIF1A* | splice-donor variant | HIGH | 9.120 | 3.231 | 0.562 |  |

Abbreviations: *AIFM2* = Apoptosis inducing factor mitochondria associated 2; *CARMIL1* = Capping protein regulator and myosin 1 linker 1; *MFGE8* = Milk fat globule EGF factor 8; *YIF1A* = Yip1 interacting factor homolog A.

^1^Novel isoforms co-localised with the SNV genomic region and are therefore predicted to be potentially affected by the SNV; however, these isoforms are not currently annotated in Ensembl and could not be evaluated by the Variant Effect Predictor (VEP).

^2^Gene expression is given in FPKM (fragments per kilobase per million mapped reads), calculated to normalize read counts per library size and gene length.

^3^FDR = False Discovery Rate. Differentially expressed (DE) mRNA isoforms are indicated in bold (FDR < 0.05) and (–) indicates not reported due to low or absent expression.

**Supplementary Table S6.** Main single nucleotide variants (SNVs) leading to splice site effect (SSE) located in genes with differentially expressed mRNA isoforms in A2A2 β-casein group of cows.

| **A2A2 associated SNV ID** | **Transcript** | **Gene** | **Consequence** | **Impact** | **A1A1 Average FPKM^2^** | **A2A2 Average FPKM^2^** | **FDR^3^** | **Main Molecular Function** |
| --- | --- | --- | --- | --- | --- | --- | --- | --- |
| **Secretory transport and membrane trafficking** | | | | | | | | |
| **-** | **ABCG2_1** | ***ABCG2*** | **-** | **-** | **0.000** | **2.644** | **0.023** | Apical transport to milk |
| - | ABCG2_2 | *ABCG2* |  | - | 25.742 | 36.809 | 0.871 |  |
| - | ABCG2_3^1^ | *ABCG2* | - | - | 0.721 | 1.768 | 0.957 |  |
| - | ABCG2_4^1^ | *ABCG2* | - | - | 0.999 | 1.914 | 0.810 |  |
| 6_36566686_G/A | ABCG2-202 | *ABCG2* | splice-acceptor variant | HIGH | 4.216 | 5.885 | 0.970 |  |
| 6_36566686_G/A | ABCG2-204 | *ABCG2* | splice-acceptor variant | HIGH | 122.842 | 189.874 | 0.675 |  |
| **8_111160250_G/T** | **EIPR1-203** | ***EIPR1*** | **splice-donor variant** | **HIGH** | **1.915** | **0.000** | **0.002** | Endosomal trafficking |
| **Mitochondrial gene expression** | | | | | | | | |
| - | NSUN3_3^1^ | *NSUN3* | - | - | 0.000 | 0.334 | 0.040 | Mitochondrial protein synthesis |
| - | NSUN3_4^1^ | *NSUN3* | - | - | 0.371 | 0.187 | 0.796 |  |
| - | NSUN3_5^1^ | *NSUN3* | - | - | 1.274 | 1.500 | 0.999 |  |
| **-** | **NSUN3_6^1^** | ***NSUN3*** | **-** | **-** | **0.000** | **0.385** | **0.035** |  |
| 1_38360664_G/C | NSUN3-201 | *NSUN3* | splice-donor variant | HIGH | 0.270 | 0.277 | 0.998 |  |
| **Epigenetic and transcriptional regulation** | | | | | | | | |
| - | EHMT2_1^1^ | *EHMT2* | - | - | 0.551 | 1.236 | 0.817 | Epigenetic repression |
| - | EHMT2_2^1^ | *EHMT2* | - | - | 1.465 | 2.533 | 0.887 |  |
| 23_27468625_G/A | EHMT2-201 | *EHMT2* | splice-donor variant | HIGH | 0.436 | 0.678 | 0.981 |  |
| **23_27468625_G/A** | **EHMT2-202** | ***EHMT2*** | **splice-donor variant** | **HIGH** | **0.287** | **0.006** | **0.038** |  |
| 23_27468625_G/A | EHMT2-203 | *EHMT2* | splice-donor variant | HIGH | 1.617 | 0.718 | 0.748 |  |

Abbreviations: *ABCG2* = ATP binding cassette subfamily G member 2*; EIPR1* = Endosome integrity protein related 1; *NSUN3* = NOP2/Sun RNA methyltransferase family member 3; *EHMT2* = Euchromatic histone lysine methyltransferase.

^1^Novel isoforms co-localised with the SNV genomic region and are therefore predicted to be potentially affected by the SNV; however, these isoforms are not currently annotated in Ensembl and could not be evaluated by the Variant Effect Predictor (VEP).

^2^Gene expression is given in FPKM (fragments per kilobase per million mapped reads), calculated to normalize read counts per library size and gene length.

^3^FDR = False Discovery Rate. Differentially expressed (DE) mRNA isoforms are indicated in bold (FDR < 0.05) and (–) indicates not reported due to low or absent expression.

**Supplementary Table S7.** Theoretical VEP annotations of non-expressed transcripts overlapping genomic positions of SNVs with predicted splice site effects (SSE) detected in expressed transcripts of A1A1 β-casein cows.

| **A1A1 associated SNV ID** | **Transcript** | **Gene** | **Consequence** | **Impact** | **A1A1 Average FPKM^2^** | **A2A2 Average FPKM^2^** | **FDR^3^** | **Main Molecular Function** |
| --- | --- | --- | --- | --- | --- | --- | --- | --- |
| **Mitochondrial redox balance and cell survival** | | | | | | | | |
| - | AIFM2_2^1^ | *AIFM2* | - | - | 0.039 | 0.073 | 0.961 | Mitochondrial redox control and ferroptosis inhibition |
| - | AIFM2_3^1^ | *AIFM2* | - | - | 0.306 | 0.051 | 0.457 |  |
| - | AIFM2_6^1^ | *AIFM2* | - | - | 0.078 | 0.068 | 0.991 |  |
| - | AIFM2_7^1^ | *AIFM2* | - | - | 0.098 | 0.058 | 0.939 |  |
| 28_26347419_A/G | AIFM2-202 | *AIFM2* | splice-donor variant | HIGH | 0.156 | 0.025 | 0.412 |  |
| **Cytoskeleton organisation and secretion mechanics** | | | | | | | | |
| 23_32397444_T/C | CARMIL1-202 | *CARMIL1* | missense variant, splice-region variant | MODERATE/SIFT 0 | 0.010 | 0.066 | 0.558 | Actin cytoskeleton dynamics |
| 23_32397444_T/C | CARMIL1-205 | *CARMIL1* | missense variant, splice-region variant | MODERATE/SIFT 0 | 0.033 | 0.188 | 0.572 |  |
| 23_32397444_T/C | CARMIL1-206 | *CARMIL1* | missense variant, splice-region variant | MODERATE/SIFT 0 | 0.084 | 0.131 | 0.986 |  |
| **Milk fat globule (MFG) structure and secretion** | | | | | | | | |
| - | MFGE8_4 | *MFGE8* | - | - | 0.050 | 0.036 | - | Milk fat globule membrane (MFGM) component |
| 21_20476454_C/T | MFGE8-202 | *MFGE8* | splice-donor variant | HIGH | 0.028 | 0.047 | 0.975 |  |
| 21_20476454_C/T | MFGE8-203 | *MFGE8* | splice-donor variant | HIGH | 0.000 | 0.000 | - |  |
| 21_20476454_C/T | MFGE8-205 | *MFGE8* | splice-donor variant | HIGH | 0.020 | 0.000 | - |  |
| **Vesicular trafficking** | | | | | | | | |
| - | YIF1A_1 | *YIF1A* | - | - | 0.000 | 0.126 | 0.200 | Golgi vesicular trafficking |
| 29_44342558_C/G | YIF1A-202 | *YIF1A* | splice-donor variant | HIGH | 0.183 | 0.000 | 0.111 |  |
| 29_44342558_C/G | YIF1A-203 | *YIF1A* | splice-donor variant | HIGH | 0.000 | 0.006 | - |  |

Abbreviations: *AIFM2* = Apoptosis inducing factor mitochondria associated 2; *CARMIL1* = Capping protein regulator and myosin 1 linker 1; *MFGE8* = Milk fat globule EGF factor 8; *YIF1A* = Yip1 interacting factor homolog A.

^1^Novel isoforms co-localised with the SNV genomic region and are therefore predicted to be potentially affected by the SNV; however, these isoforms are not currently annotated in Ensembl and could not be evaluated by the Variant Effect Predictor (VEP).

^2^Gene expression is given in FPKM (fragments per kilobase per million mapped reads), calculated to normalize read counts per library size and gene length.

^3^FDR = False Discovery Rate. Differentially expressed (DE) mRNA isoforms are indicated in bold (FDR < 0.05) and (–) indicates not reported due to low or absent expression.

**Supplementary Table S8.** Theoretical VEP annotations of non-expressed transcripts overlapping genomic positions of SNVs with predicted splice site effects (SSE) detected in expressed transcripts of A2A2 β-casein cows.

| **A2A2 associated SNV ID** | **Transcript** | **Gene** | **Consequence** | **Impact** | **A1A1 Average FPKM^2^** | **A2A2 Average FPKM^2^** | **FDR^3^** | **Main Molecular Function** |
| --- | --- | --- | --- | --- | --- | --- | --- | --- |
| **Secretory transport and membrane trafficking** | | | | | | | | |
| 6_36566686_G/A | ABCG2-203 | *ABCG2* | splice-acceptor variant | HIGH | 0.000 | 0.000 | - | Apical transport to milk |
| 8_111160250_G/T | EIPR1-201 | *EIPR1* | downstream gene variant | MODIFIER | 0.000 | 0.133 | 0.148 | Endosomal trafficking |
| 8_111160250_G/T | EIPR1-202 | *EIPR1* | splice-donor variant | HIGH | 0.025 | 0.044 | 0.982 |  |
| 8_111160250_G/T | EIPR1-204 | *EIPR1* | synonymous variant | LOW | 0.000 | 0.134 | 0.149 |  |
| **Mitochondrial energy metabolism (oxidative phosphorylation, OXPHOS)** | | | | | | | | |
| - | ATP5MG_1 | *ATP5MG* | - | - | 0.019 | 0.000 | - | Mitochondrial OXPHOS (ATP synthase) |
| 15_29103552_C/T | ATP5MG-202 | *ATP5MG* | missense variant, splice-region variant | MODERATE | 0.066 | 0.123 | 0.952 |  |
| - | ATP5MG-203 | *ATP5MG* | - | - | 0.117 | 0.068 | - |  |
| - | ATP5MG-204 | *ATP5MG* | - | - | 0.000 | 0.000 | - |  |
| **Mitochondrial gene expression** | | | | | | | | |
| - | NSUN3_1^1^ | *NSUN3* | - | - | 0.137 | 0.050 | 0.792 | Mitochondrial protein synthesis |
| - | NSUN3_2^1^ | *NSUN3* | - | - | 0.077 | 0.059 | 0.982 |  |
| - | NSUN3_7^1^ | *NSUN3* | - | - | 0.000 | 0.034 | 0.252 |  |
| **Mitochondrial integrity and dinamics** | | | | | | | | |
| 7_109738547_C/T | SLC25A46-201 | *SLC25A46* | intron variant | MODIFIER | 0.000 | 0.000 | - | Mitochondrial integrity and dinamics |
| 7_109738547_C/T | SLC25A46-202 | *SLC25A46* | splice-region variant, synonymous variant | LOW | 0.000 | 0.000 | - |  |
| 7_109738547_C/T | SLC25A46-203 | *SLC25A46* | splice-region variant, synonymous variant | LOW | 0.000 | 0.002 | - |  |
| - | SLC25A46-204 | *SLC25A46* | - | - | 0.018 | 0.157 | 0.351 |  |

Abbreviations: *ABCG2* = ATP binding cassette subfamily G member 2*; EIPR1* = Endosome integrity protein related 1; *ATP5MG* = ATP synthase membrane subunit g; *NSUN3* = NOP2/Sun RNA methyltransferase family member 3; *SLC25A46* = Solute carrier family 25 member 46; *EHMT2* = Euchromatic histone lysine methyltransferase ^1^Novel isoforms co-localised with the SNV genomic region and are therefore predicted to be potentially affected by the SNV; however, these isoforms are not currently annotated in Ensembl and could not be evaluated by the Variant Effect Predictor (VEP).

^2^Gene expression is given in FPKM (fragments per kilobase per million mapped reads), calculated to normalize read counts per library size and gene length.

^3^FDR = False Discovery Rate. Differentially expressed (DE) mRNA isoforms are indicated in bold (FDR < 0.05) and (–) indicates not reported due to low or absent expression.
